# Certified randomness using a trapped-ion quantum processor

**DOI:** 10.1038/s41586-025-08737-1

**Published:** 2025-03-26

**Authors:** Minzhao Liu, Ruslan Shaydulin, Pradeep Niroula, Matthew DeCross, Shih-Han Hung, Wen Yu Kon, Enrique Cervero-Martín, Kaushik Chakraborty, Omar Amer, Scott Aaronson, Atithi Acharya, Yuri Alexeev, K. Jordan Berg, Shouvanik Chakrabarti, Florian J. Curchod, Joan M. Dreiling, Neal Erickson, Cameron Foltz, Michael Foss-Feig, David Hayes, Travis S. Humble, Niraj Kumar, Jeffrey Larson, Danylo Lykov, Michael Mills, Steven A. Moses, Brian Neyenhuis, Shaltiel Eloul, Peter Siegfried, James Walker, Charles Lim, Marco Pistoia

**Affiliations:** 1 Global Technology Applied Research, JPMorganChase, New York, NY USA; 2 Computational Science Division, Argonne National Laboratory, Lemont, IL USA; 3 Department of Physics, The University of Chicago, Chicago, IL USA; 4 Quantinuum, Broomfield, CO USA; 5 Department of Computer Science, The University of Texas at Austin, Austin, TX USA; 6 Department of Electrical Engineering, National Taiwan University, Taipei City, Republic of China; 7 Quantinuum, Terrington House, Cambridge, UK; 8 Quantum Science Center, Oak Ridge National Laboratory, Oak Ridge, TN USA; 9 Mathematics and Computer Science Division, Argonne National Laboratory, Lemont, IL USA; 10 Present Address: NIVIDA Corporation, Santa Clara, CA USA

**Keywords:** Quantum information, Computer science, Qubits

## Abstract

Although quantum computers can perform a wide range of practically important tasks beyond the abilities of classical computers^1,2^, realizing this potential remains a challenge. An example is to use an untrusted remote device to generate random bits that can be certified to contain a certain amount of entropy^3^. Certified randomness has many applications but is impossible to achieve solely by classical computation. Here we demonstrate the generation of certifiably random bits using the 56-qubit Quantinuum H2-1 trapped-ion quantum computer accessed over the Internet. Our protocol leverages the classical hardness of recent random circuit sampling demonstrations^4,5^: a client generates quantum ‘challenge’ circuits using a small randomness seed, sends them to an untrusted quantum server to execute and verifies the results of the server. We analyse the security of our protocol against a restricted class of realistic near-term adversaries. Using classical verification with measured combined sustained performance of 1.1 × 10^18^ floating-point operations per second across multiple supercomputers, we certify 71,313 bits of entropy under this restricted adversary and additional assumptions. Our results demonstrate a step towards the practical applicability of present-day quantum computers.

## Abstract (web summary)

Certifiably random bits can be generated using the 56-qubit Quantinuum H2-1 trapped-ion quantum computer accessed over the Internet.

## Main

In recent years, numerous theoretical results have shown evidence that quantum computers have the potential to tackle a wide range of problems out of reach of classical techniques. The main examples include factoring large integers^6^, implicitly solving exponentially sized systems of linear equations^7^, optimizing intractable problems^8^, learning certain functions^9^ and simulating large quantum many-body systems^10^. However, accounting for considerations such as quantum error correction overheads and gate speeds, the resource requirements of known quantum algorithms for these problems put them far outside the reach of near-term quantum devices, including many suggested fault-tolerant architectures. Consequently, it is unclear whether the devices available in the near term can benefit a practical application^11^.

Starting with one of the first ‘quantum supremacy’ demonstrations^5^, several groups have used random circuit sampling (RCS) as an example of a task that can be executed faster and with a lower energy cost on present-day quantum computers compared with what is achievable classically^4,12–14^. Yet, despite rapid experimental progress, a beyond-classical demonstration of a practically useful task performed by gate-based quantum computers has so far remained unknown.

Random number generation is a natural task for the beyond-classical demonstration because randomness is intrinsic to quantum mechanics, and it is important in many applications, ranging from information security to ensuring the fairness of processes such as jury selection^15–17^. The main challenge for any client receiving randomness from a third-party provider, such as a hardware security module, is to verify that the bits received are truly random and freshly generated. Although certified randomness is not necessary for every use of random numbers, the freshness requirement is especially important in applications such as lotteries and e-games, in which several parties (which may or may not trust each other) need to ensure that a publicly distributed random number was generated on demand. Moreover, certified randomness can be used to verify the position of a dishonest party^18–20^.

Protocols exist for certifying random numbers based on the violation of Bell inequalities^15,21–24^. However, these protocols typically require the underlying Bell test to be loophole-free, which can be hard for the client to enforce when the quantum devices are controlled by a third-party provider. This approach thus necessitates that the client trust a third-party quantum device provider to perform the Bell test faithfully.

**Fig. 1:**
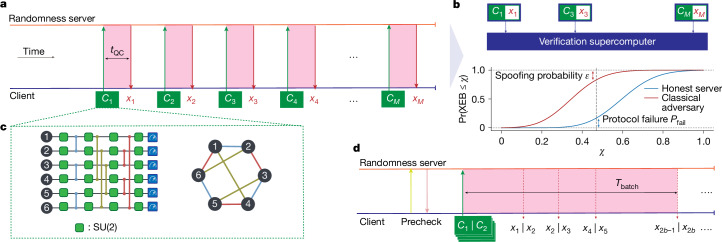
Overview of the protocol. **a**, The idealized protocol. A client submits *M* random circuits ${\{{C}_{i}\}}_{i\in [M]}$ serially to a randomness server and expects bitstrings ${\{{x}_{i}\}}_{i\in [M]}$ back, each within a time *t*_QC_. **b**, A subset of circuit-bitstring pairs is used to compute the XEB score. The XEB score has distributions (bottom plot for qualitative illustration only) corresponding to either an honest server or an adversarial server performing a low-fidelity classical simulation. For any XEB target indicated by the dashed line, an honest server may fail to achieve a score above this threshold with a probability *P*_fail_. **c**, Illustration of the challenge circuits, consisting of layers of *U*_ZZ_ gates sandwiched between layers of random SU(2) gates on all qubits. The arrangement of two-qubit gates is obtained via edge colouring (right) on a random *n*-node graph. **d**, Client-server interaction as implemented in our protocol. Following a device-readiness check (‘precheck’), the client submits a batch of 2*b* circuits and expects all the samples corresponding to the batch to be returned within a cutoff duration *T*_*b*,cutoff_. Note that only one batch with execution time *T*_batch_ is illustrated in the figure. The client continues the protocol until *M* total circuits have been successfully executed.

Alternatively, ref. ^3^ proposed a certified randomness protocol that combines RCS with ‘verification’ on classical supercomputers^3,25^. This type of protocol allows a classical client to verify randomness using only remote access to an untrusted quantum server. A classical client pseudorandomly generates *n*-qubit challenge circuits and sends them to a quantum server, which is asked to return length-*n* bitstrings sampled from the output distribution of these circuits within a short amount of time (Fig. 1a,c). The circuits are chosen such that no realistic adversarial server can classically simulate them within the short response time. A small subset of circuits is then used to compute the cross-entropy benchmarking (XEB) score^26^ (Fig. 1b), which reflects how well the samples returned by the server match the ideal output distributions of the submitted circuits. Extensive complexity-theoretic evidence suggests that XEB is hard to ‘spoof’ classically^27,28^. Therefore, a high XEB score, combined with a short response time, allows the client to certify that the server must have used a quantum computer to generate its responses, thereby guaranteeing a certain amount of entropy with high probability. Our analysis quantifies the minimum amount of entropy that an untrusted server, possibly acting as an adversary, must provide to achieve a given XEB score in a short amount of time.

The protocol proposed in ref. ^3^ provides a complexity-theoretic guarantee of *Ω*(*n*) bits of entropy for a server returning many samples from the same circuit. This protocol is best suited for quantum computing architectures with overheads that make it preferable to sample a circuit many times after loading it once. In practice, the classical simulation cost of sampling a circuit many times is comparable to the cost of sampling only once^29^. Furthermore, the trapped-ion-based quantum computer used in this work is configured to feature minimal overhead per circuit, such that executing many single-shot circuits does not introduce a substantial time penalty per circuit compared with sampling one circuit many times. Together, these two observations motivate strengthening the security of the protocol by requesting the server to return only one sample per circuit. To this end, in Supplementary Information section I, we extend the complexity-theoretic analysis to this modified setting of one sample per circuit, guaranteeing *Ω*(*n*) bits of entropy.

In this work, we report an experimental demonstration of an RCS-based certified randomness protocol. Our main contributions are as follows. First, inspired by ref. ^3^, we propose a modified RCS-based certified randomness protocol that is tailored to near-term quantum servers. Second, we prove the security of our implementation against a class of realistic finite-sized adversaries. Third, we use a high-fidelity quantum computer and exascale classical computation to experimentally realize this proposed protocol, pushing the boundaries of both quantum and classical computing abilities. By combining the high-fidelity Quantinuum H2-1 quantum processor with exascale verification, we demonstrate a useful beyond-classical application of gate-based digital quantum computers.

In our proposed protocol, shown in Fig. 1d and detailed in the Methods, the client pseudorandomly generates a sufficiently large number of *n*-qubit quantum circuits and then sends them in batches of 2*b* circuits, where *b* is an integer. After a batch is submitted, the client waits for 2*b* length-*n* bitstrings to be returned within *T*_*b*,cutoff_ seconds. The batch cutoff time prevents the protocol from stalling and is fixed in advance based on preliminary experiments to a value intended to maximize the amount of certifiable entropy while ensuring that the average response time per circuit remains low enough to preclude classical simulation as a viable strategy for the server to generate responses. If a batch times out or if a failure status is reported, all of the outstanding jobs in the batch are cancelled, and all bitstrings received from the batch are discarded. Consequently, results from a failed batch are not included in calculating the XEB score or entropy extraction. Batches are continually submitted until *M* valid samples are collected. The cumulative response time for successful batches gives the total time *T*_tot_ and the average time per sample *t*_QC_ = *T*_tot_/*M*. Subsequently, the client calculates the XEB score on a subset of size *m* randomly sampled from the *M* circuit–sample pairs:

1 $${{\rm{XEB}}}_{{\rm{test}}}=\frac{{2}^{n}}{m}\sum _{i\in {\mathcal{V}}}{P}_{{C}_{i}}({x}_{i})-1,$$

where ${\mathcal{V}}$ is the set of indices for the random subset of size *m* and *P*_*C*_(*x*) = |⟨*x*|*C*|0⟩|^2^ is the probability of measuring bitstring *x* from an ideal quantum computer executing circuit *C*. If the bitstrings *x*_*i*_ are perfectly drawn from the output distributions of sufficiently deep random circuits *C*_*i*_, the XEB score is expected to concentrate around 1. By contrast, if the *x*_*i*_ are drawn from distributions uncorrelated with the distributions induced by *C*_*i*_, the XEB score is expected to concentrate around 0. The client decides to accept the received samples as random bits based on two criteria. First, the average time per sample must be lower than a threshold *t*_threshold_, which is chosen to preclude high-fidelity classical simulation. This time can be lower than *T*_*b*,cutoff_ because it is advantageous from the perspective of extractable entropy to accept some samples with response time slightly larger than *t*_threshold_ as long as the average response time remains low. Second, the XEB score on ${\mathcal{V}}$ must be greater than a threshold *χ* ∈ [0, 1]. All of *t*_threshold_, *χ* and *T*_*b*,cutoff_ are determined in advance of protocol execution, based on (for example) preliminary hardware experiments, with the goal of certifying a certain fixed amount of entropy at the end of the protocol with high probability. Together, the protocol succeeds if

2 $${t}_{{\rm{QC}}}={T}_{{\rm{tot}}}/M\le {t}_{{\rm{threshold}}}\quad \,\text{and}\,\,\,{{\rm{XEB}}}_{{\rm{test}}}\ge \chi ,$$

and otherwise aborts.

**Fig. 2:**
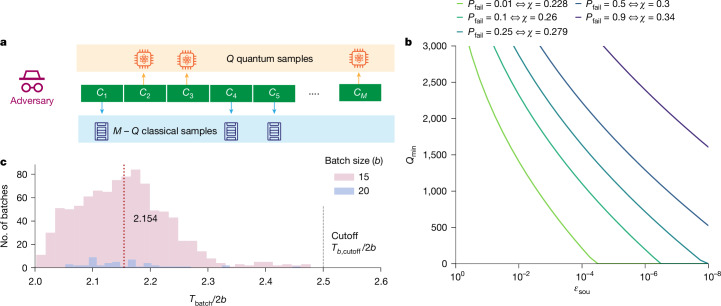
Adversary model and protocol security. **a**, In the adversarial model considered in this work, *Q* samples are obtained using a perfect-fidelity quantum computer and *M* − *Q* using classical simulation. **b**, Probability of an honest server with fidelity *ϕ* = 0.3 failing to certify *Q*_min_ quantum samples (and corresponding threshold *χ*) with soundness *ε*_sou_ against an adversary four times more powerful than Frontier over repeated experiments, with the protocol parameters set to those from Table 1. **c**, Distribution of batch times per successful sample, from a total of 984 successful batches, in our experiment. The vertical dashed line indicates the average time per sample.

The security of our protocol relies on the central assumption that, for the family of pseudorandom circuits we consider, there exists no practical classical algorithm that can spoof the XEB test used in the protocol. We analyse the protocol security by modelling a restricted but realistic adversarial server that we believe to be the most relevant: for each circuit received, the adversary either samples an output honestly from a quantum computer or performs classical simulation (Fig. 2a). As only the former contains entropy, the adversary tries to achieve the threshold XEB score with the fewest quantum samples, to pass the XEB test while returning as little entropy as possible. For our protocol, we assume an adversary with a perfect-fidelity quantum computer, which allows the adversary to spoof the maximum number of bitstrings classically. We further assume that the classical computational power of the adversary is bounded by a fixed number of floating-point operations per second (FLOPS) ${\mathcal{A}}$, which may be measured relative to the most powerful supercomputer in the world (at the time of experiment, the Frontier supercomputer; see https://www.top500.org/lists/top500/2024/06/), and that the adversary possesses the same optimized methods to simulate the circuits as the client has. Note that an adversary possessing more powerful classical methods for simulating circuits than expected can equivalently be modelled as an adversary with identical classical methods and larger computational power. We note that as the adversaries we analyse are allowed only a restricted set of strategies, the subsequent mathematical results hold only in this limited setting, conditioned on some additional assumptions further detailed in Supplementary Information section IIIC. To the best of our knowledge, the restricted set of classical and quantum adversary strategies considered here correspond to the current state of the art. We leave the incorporation of a broader class of adversaries to future analysis.

The client needs to ensure that the circuits are difficult to simulate within the time *t*_threshold_. Otherwise, the server can use its classical supercomputer to deterministically simulate the circuits with high fidelity and generate samples that readily pass the tests in equation (2). For the family and size of circuits we consider, tensor network contraction is the most performant known method for finite-fidelity and exact simulation^4^ as well as sampling. If a circuit has a verification (exact simulation) cost of ${\mathcal{B}}$ FLOPS, the adversary can simulate each circuit to a target fidelity of ${\mathcal{A}}\cdot {t}_{{\rm{threshold}}}/{\mathcal{B}}$ using partial contraction of tensor networks, for which the simulation cost and simulation fidelity are related linearly^30^. The protocol is successful only if the parameters are chosen such that the fidelity *ϕ* of an honest server satisfies

3 $$\phi \gg {\mathcal{A}}\cdot {t}_{{\rm{threshold}}}/{\mathcal{B}}.$$

This condition requires that there exists a gap between the fidelity of an honest server and that achievable by an adversary performing mostly classical simulations. If this condition is satisfied, the XEB score of an honest server will have a probability distribution with a higher average value than the probability distribution of the XEB of the adversary (qualitatively shown in Fig. 1b), allowing the client to distinguish between the two.

After certification (that is, if the tests in equation (2) pass), the client uses a randomness extractor to process the *M* samples. An ideal protocol for certified randomness either aborts, resulting in an ‘abort state’, or succeeds, resulting in a uniformly distributed bitstring that is uncorrelated with any side information. Viewing the protocol as a channel acting on some initial state composed of both the server and the client, an end-to-end protocol is said to be *ε*_sou_-sound if, for any initial state, the end result is *ε*_sou_-close (in terms of trace distance) to the ideal output: a mixture of the abort state and the maximally mixed state (see Supplementary Information section IIIA for the rigorous definition of soundness).

The entropy that the client can extract out of the received samples on successful execution of the protocol depends on how stringent its thresholds on the response time (*t*_threshold_) and the XEB score (*χ*) are. It is in the interest of the client to set these thresholds as stringently as possible, to force the hypothetical adversary to draw more samples from the quantum computer, while still allowing that an honest server can succeed with high probability. As the thresholds are known to both parties, the strategy of the adversary is to minimize the use of the quantum computer while ensuring that the protocol does not abort. Based on the protocol thresholds, the client can determine the number of quantum samples *Q*_min_ such that the protocol aborts with a large probability 1 − *ε*_accept_ if the adversary returns fewer than *Q*_min_ samples from the quantum computer (see Supplementary Information section IVF for details). This lower bound on *Q*_min_ can be used to derive the minimum smooth min-entropy of the received samples. Note that the smooth min-entropy of an information source characterizes the number of random bits that can be extracted from the source. In particular, we devise an *ε*_sou_-sound protocol that provides a lower bound on the smooth min-entropy ${H}_{\min }^{{\varepsilon }_{{\rm{s}}}}$ (defined in Supplementary Information section IIID) with smoothness parameter *ε*_s_ = *ε*_sou_/4 and with *ε*_accept_ = *ε*_sou_. The results in the paper are reported in terms of the soundness parameter *ε*_sou_ and the smooth min-entropy ${H}_{\min }^{{\varepsilon }_{{\rm{s}}}}$.

A smaller *ε*_sou_ makes a stronger security guarantee by making it more difficult for an adversary to pass the XEB test with a small *Q*_min_. This may be achieved by choosing a higher threshold *χ*. However, a higher threshold also makes it more likely for an honest server to fail the XEB test, meaning that the honest server cannot be certified to have produced the target amount of extractable entropy. Note that this does not necessarily mean that the samples provided by the honest server do not contain entropy, only that they fail to satisfy the criteria of equation (2) and consequently the protocol aborts. In practice, it is desirable to ensure that an honest server fails only with a low failure probability *P*_fail_. To that end, we may compute a threshold *χ*(*P*_fail_) corresponding to any acceptable *P*_fail_. This threshold, along with *t*_threshold_, then allows us to determine *Q*_min_ for a target soundness *ε*_sou_ (Supplementary Information section IIID). Figure 2b shows the achievable *Q*_min_ at different *P*_fail_ and *ε*_sou_, showing the trade-off between the three quantities at the fixed experimental configuration and the classical computational power of adversary ($\phi ,{t}_{{\rm{QC}}},M,m,{\mathcal{B}}$ and ${\mathcal{A}}$).

We demonstrate our protocol using the Quantinuum H2-1 trapped-ion quantum processor accessed remotely over the Internet. The experimental parameters are provided in Table 1. The challenge circuits (shown in Fig. 1c, see Supplementary Information section IVC for the considerations involved in choosing the circuits) have a fixed arrangement of 10 layers of entangling *U*_ZZ_ gates, each sandwiched between layers of pseudorandomly generated *SU*(2) gates on all qubits. The arrangement of two-qubit gates is obtained by edge colouring on a random *n*-node graph. Preliminary mirror-benchmarking experiments, along with gate-counting arguments based on the measured fidelities of component operations, enable us to estimate the fidelity of an honest server^4^. At the time of the experiment, the H2-1 quantum processor was expected to attain a fidelity of *ϕ* ≳ 0.3 or better on depth-10 circuits (multiple improvements were made to the H2-1 device after the collection of the data of this experiment that slightly increased the fidelity estimate in ref. ^4^). Likewise, the same preliminary experiments also let us anticipate average time per sample to be approximately 2.1 s, with a long-tailed timing distribution out to just below 2.5 s, as also seen in the full experiment in Fig. 2c. Reasonable (*P*_fail_ = 50%) protocol success rates can therefore be achieved with thresholds *t*_threshold_ = 2.2 s and *χ* = 0.3. For illustrative purposes, we describe the experiment based on these choices (in practice, one might want to lower *P*_fail_ by setting *χ* somewhat below the expected value). The batch cutoff time is set to be *T*_*b*,cutoff_ = (2*b*) × 2.5 s, anticipating that the relatively small expected fraction of batches taking average time per sample between *t*_threshold_ = 2.2 s and 2.5 s would contribute additional entropy to the received samples while being unlikely to increase the average time per sample from the expected 2.1 s past the threshold of 2.2 s.

**Table 1:** Summary of experimental parameters

Label	Meaning	Value
*n*	Number of qubits	56
${\mathcal{B}}$	Cost of simulating challenge circuits	90 × 10^18^ FLOPS
${\mathcal{A}}$	Sustained peak performance of the Frontier supercomputer	0.897 × 10^18^ FLOPS
–	Time to simulate challenge circuits on the Frontier supercomputer	100.3 s
*χ*	Threshold for XEB test	0.3
*t*_threshold_	Threshold for average time per sample	2.2 s
*T*_*b*,cutoff_	Cutoff time for the server to respond to a batch of 2*b* circuits	2.5 × 2*b* s
*M*	Number of successful samples	30,010
*t*_QC_	Average response time per successful quantum sample	2.154 s
*m*	Number of samples used to measure XEB	1,522
XEB_test_	Measured XEB	0.32

The circuit family considered has a simulation cost of ${\mathcal{B}}=90\times 1{0}^{18}$ FLOPS on the Frontier supercomputer of the Department of Energy^31^, the most powerful supercomputer in the world, to our knowledge, at the time of writing (https://www.top500.org/lists/top500/2024/06/). Following a detailed estimate of runtime on Frontier, we determine an exact simulation time of 100.3 s per circuit when using the entire supercomputer at a numerical efficiency of 45%, where numerical efficiency is the ratio between the actual algorithm runtime and its theoretical expectation (see Supplementary Information section IVA for details on the circuit simulation cost).

In our experiment, we use two batch sizes, *b* = 15 and *b* = 20; most of the batches have *b* = 15. In total, we submitted 1,993 batches for a total of 60,952 circuits. From those, we obtain a total of *M* = 30,010 valid samples out of 984 successful batches. The cumulative device time of the successful samples was 64,652 s, giving an average time of *t*_QC_ = 2.154 s per sample, inclusive of all overheads such as communication time. Figure 2c shows the distribution of *t*_QC_ per successful sample.

In this work, the classical computational budget of the client is spread across the Frontier^31^, Summit^32^, Perlmutter^33^ and Polaris^34^ supercomputers equipped with graphics processing units (GPUs), which are especially suitable for quantum circuit simulations. Of the four supercomputers, Frontier and Summit were used at full-machine scale during verification. We measure the sustained peak performance of 897 petaFLOPS and 228 petaFLOPS, respectively (corresponding to numerical efficiencies of 45% and 59%), achieving a combined performance of 1.1 exaFLOPS (see Supplementary Information section IVE). We compute the XEB score for *m* = 1,522 circuit–sample pairs, obtaining XEB_test_ = 0.32. The complete set of experimental parameters is listed in Table 1.

The measured fidelity of XEB_test_ = 0.32 and measured time per sample *t*_QC_ = 2.154 s pass the protocol specified by *χ* = 0.3 and *t*_threshold_ = 2.2 s. For a choice of soundness parameter *ε*_sou_ and a smoothness parameter *ε*_s_ = *ε*_sou_/4, the protocol thresholds determine the number of quantum samples *Q* and the smooth min-entropy ${H}_{\min }^{{\varepsilon }_{{\rm{s}}}}$ guaranteed by the success of this protocol against an adversary with classical resources bounded by ${\mathcal{A}}$. In Table 2, we report the smooth min-entropy rate, ${H}_{\min }^{{\varepsilon }_{{\rm{s}}}}/(56\times M)$, for a range of ${\mathcal{A}}$ and *ε*_sou_ (see Supplementary Information section IVF for details of this calculation). This is to show that if we want to increase the security of the protocol either by increasing the assumed classical computational power of the adversary or by reducing the soundness parameter, the amount of entropy that we can obtain must reduce. In particular, we highlight that at *ε*_sou_ = 10^−6^, we have *Q*_min_ = 1,297, corresponding to ${H}_{\min }^{{\varepsilon }_{{\rm{s}}}}=\mathrm{71,313}$ against an adversary four times more powerful than Frontier (under the assumptions discussed earlier).

**Table 2:** Smooth min-entropy rate at varying *ε*_sou_ and $\boldsymbol{\mathcal{A}}$

	$\boldsymbol{\mathcal{A}}$ (multiples of Frontier)				
*ε*_sou_	1	2	4	6	8
10^−2^	0.19	0.16	0.11	0.06	0.01
10^−4^	0.15	0.12	0.07	0.02	0.00
10^−6^	0.12	0.09	**0.04**	0.00	0.00
10^−8^	0.10	0.07	0.02	0.00	0.00
10^−10^	0.08	0.05	0.00	0.00	0.00

The adversary is assumed to have the same efficiency for classical simulation as client verification. The ratio corresponding to the entropy we report in the main text is boldfaced.

We feed the 56 × 30,010 raw bits into a Toeplitz randomness extractor^35^ and extract 71,273 bits (see Supplementary Information section IVF for details on extraction and the determination of extractable entropy). We note that the Toeplitz extractor is a ‘strong’ seeded extractor for which the output is independent of the seed. For private use of the randomness, in which the extracted bits are not shown, the extractor seed can be reused. We append the seed used in the extractor to the protocol output and do not count the seed as randomness ‘consumed’ by our protocol. The total input randomness used to seed the pseudorandom generator is thereby only 32 bits, and our protocol achieves certified randomness expansion. We further note that other extractors can be used that may consume less seed but have different security guarantees.

**Fig. 3:**
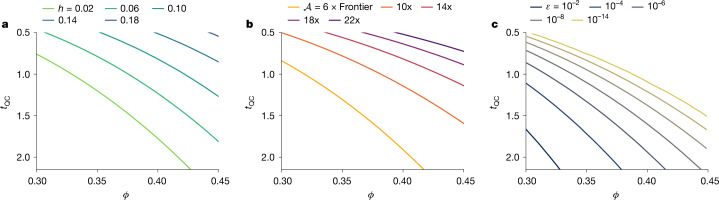
Future improvements. Improvement in metrics as fidelity *ϕ* and time per sample *t*_QC_ improve. All panels assume the same verification budget as this experiment, classical simulation numerical efficiency of 50% for both verification and spoofing, and target failure probability *P*_fail_ = 10^−4^. **a**, Smooth min-entropy rate, $h={H}_{\min }^{{\varepsilon }_{{\rm{s}}}}/(M\cdot n)$, against an adversary four times as powerful as Frontier with *ε*_sou_ = 10^−6^ and *ε*_s_ = *ε*_sou_/4. **b**, Adversarial power that still allows *h* = 0.01 to be guaranteed with *ε*_sou_ = 10^−6^. **c**, Soundness parameter *ε*_sou_ that still allows *h* = 0.01 to be guaranteed with an adversary that is four times as powerful as Frontier.

Future experiments are expected to improve device fidelity (higher *ϕ*) and execution speed (lower *t*_QC_). Adjusting protocol thresholds (*χ* and *t*_threshold_) against improved device specifications stands to improve our protocol in terms of the achievable entropy, the adversarial computational power that can be guarded against and the soundness parameter. Figure 3 shows these metrics as we improve *t*_QC_ and *ϕ* (see Supplementary Information section V for details of this calculation). Conversely, for a fixed adversary and soundness parameter, any improvement in *t*_QC_ and *ϕ* reduces the verification budget required to certify a target number of quantum samples *Q*, making our protocol more cost-effective. Any improvement in entropy, all else being equal, translates into a higher throughput in the sense of a higher rate of entropy generation per second. With *χ* = 0.3 and *t*_threshold_ = 2.2 s, our experiment has a bitrate of 71,273/(30,010 × 2.2 s) ≈ 1 bit per second at *ε*_sou_ = 10^−6^. For *ε*_sou_ = 10^−6^ and *P*_fail_ = 0.1, improving fidelity to *ϕ* = 0.67 and response time to *t*_QC_ = 0.55 s would let us achieve the bitrate of the NIST Public Randomness beacon^36^ (512 bits per minute). We note that improvement in *t*_QC_ can come from higher clock rates as well as parallelization over multiple quantum processors or over many qubits of one large quantum processor.

The security of our protocol relies on the circuits being difficult to simulate. When better exact simulation techniques are developed by researchers in the future, both the adversary and the client can use the improved techniques to spoof and verify: these symmetric gains neutralize each other. Although a notable improvement in approximate simulation techniques may benefit spoofing asymmetrically, the client might be able to neutralize those gains by modifying the ensemble of challenge circuits to make approximate simulations more difficult.

In summary, this work implements a protocol for certified randomness, which also lends itself to multiparty and public verification. We note that the bit rate and soundness parameter achieved by our experiment, the restricted adversarial model, as well as the numerous assumptions used in our analysis limit the immediate deployment of the proposed protocol in production applications. However, we numerically analyse how future developments may improve the security and cost-effectiveness of our protocol. Our experiments pave the way for new opportunities in cryptography and communication.

## Disclaimer

This paper was prepared for informational purposes with contributions from the Global Technology Applied Research Center of JPMorgan Chase. This paper is not a product of the Research Department of JPMorgan Chase or its affiliates. Neither JPMorgan Chase nor any of its affiliates makes any explicit or implied representation or warranty and none of them accept any liability in connection with this paper, including, without limitation, with respect to the completeness, accuracy, or reliability of the information contained herein and the potential legal, compliance, tax or accounting effects thereof. This document is not intended as investment research or investment advice, or as a recommendation, offer or solicitation for the purchase or sale of any security, financial instrument, financial product or service, or to be used in any way for evaluating the merits of participating in any transaction.

The submitted manuscript includes contributions from UChicago Argonne, Operator of Argonne National Laboratory (‘Argonne’). Argonne, a US Department of Energy Office of Science laboratory, is operated under contract no. DE-AC02-06CH11357. The US government retains for itself, and others acting on its behalf, a paid-up nonexclusive, irrevocable worldwide licence in said Article to reproduce, prepare derivative works, distribute copies to the public and perform publicly and display publicly, by or on behalf of the government. The Department of Energy will provide public access to these results of federally sponsored research in accordance with the DOE Public Access Plan http://energy.gov/downloads/doe-public-access-plan.

## Methods

The goal of the certified randomness protocol is to achieve two properties:

1. Randomness certification: outputs generated by the protocol should be close to unpredictable and uniformly distributed, uncorrelated with any side information the client, server, and the environment might possess.
2. Randomness expansion: the entropy the client certifies in the protocol should be larger than the entropy it consumes in generating the circuits and selecting the set for validation.

The *M* bitstrings received from the server, which we denote as *X*^*M*^, do not directly satisfy the randomness certification requirement as they are not uniformly distributed. They are passed to a randomness extractor Ext along with an extractor seed *K*_ext_, which is private to the client, to obtain the final output bits *K* that are uniformly distributed along with some side information. The possible side information we consider is any classical information possessed by the client, the server and the environment before the start of the protocol, and we denote this ‘snapshot’ of initial classical information as *I*_sn_. This snapshot includes any initial randomness possessed by the client or the server.

An ideal randomness certification protocol outputs a string of bits (in the register *K*) that is uniformly random and independent of *I*_sn_. That is to say, the ideal output of a successful randomness certification protocol is precisely *τ*_*K*_ ⊗ ${\rho }_{{I}_{{\rm{sn}}}}$, where *τ*_*K*_ is a maximally mixed state and ${\rho }_{{I}_{{\rm{sn}}}}$ is the quantum state representing any side information. If the protocol aborts, the output is expected to be abort state. We quantify the security or soundness of our protocol by the closeness (as given by a trace distance) between the ideal output and the actual output produced by the protocol. As a lower bound to the smooth min-entropy of the *M* raw samples returned by the server suffices to guarantee soundness by the use of randomness extractors, we present our main result in terms of bounds on the smooth min-entropy of the returned samples.

### Protocol details

Our primary objective in the protocol design is to minimize the time between the client submitting a quantum circuit and receiving the corresponding bitstring. As a result, our protocol is designed to mitigate the following experimental considerations:

1. There is a marked latency due to network communication and the time to load a circuit into the quantum device controls. Furthermore, there is also overhead associated with executing a circuit. To ameliorate this, instead of submitting circuits one at a time, we group the circuits into batches of 15 or 20 jobs, with each job consisting of two circuits joined by a layer of mid-circuit measurements and reset. Each batch of size *b*, therefore, consists of 2*b* circuits.
2. There is downtime associated with the device, such as during periodic calibrations. Before submitting a batch, a client probes the machine for readiness using a predetermined precheck circuit *C*_precheck_. This circuit announces the intent of the client to submit a batch of circuits and triggers any server-side maintenance if necessary.
3. To ensure that the device does not stall and to keep the average time per sample low, we demand that the entire batch be returned within a cutoff time of 2.5 × 2*b* s. If the entire batch is not received within this cutoff time, we cancel all outstanding jobs in the batch, and we discard all bitstrings received from this batch.

To formally describe our experimental protocol with all details accurately represented (including details on challenge circuits generation and randomness extraction), we present the following protocol.

#### Protocol arguments

$$\begin{array}{rcl}n\in {\mathbb{N}} & : & \text{Number of qubits}\\ d\in {\mathbb{N}} & : & \text{Circuit depth}\\ M\in {\mathbb{N}} & : & \text{Total number of samples}\\ b\in {\mathbb{N}} & : & \text{Batch size}\\ m\in {\mathbb{N}} & : & \text{Test set size}\\ {K}_{{\rm{seed}}}\in {\{0,1\}}^{r} & : & \text{Random bitstring that is private}\,\text{to client}\\ {T}_{b,{\rm{cutoff}}} & : & \text{Round-trip communication}\,\text{time threshold between the client and}\,\text{the server for a batch}\\ {t}_{{\rm{threshold}}} & : & \text{Threshold on the overall average}\,\text{time-per-sample}\\ \chi  & : & \text{Threshold for the XEB test}\\ {\rm{Ext}}: & : & (\kappa ,{\varepsilon }_{{\rm{ext}}})\text{-Quantum-proof strong}\,\text{extractor (see definition 4 in Supplementary Information)}\\ {K}_{{\rm{ext}}}\in {\{0,1\}}^{s} & : & {\rm{A}}\,{\rm{random}}\,{\rm{seed}}\,{\rm{for}}\,{\rm{the}}\,{\rm{extractor}}\\ {C}_{{\rm{precheck}}} & : & {\rm{A}}\,{\rm{predetermined}}\, \mbox{`} {\rm{precheck}}\mbox{'}\,{\rm{instruction}}\,{\rm{used}}\,{\rm{to}}\,{\rm{announce}}\,{\rm{the}}\,{\rm{client}}\mbox{'}{\rm{s}}\,{\rm{readiness}}\,{\rm{to}}\,{\rm{submit}}\,{\rm{a}}\,{\rm{batch}}\end{array}$$

#### Protocol steps

1. Set the samples collected ${{\mathcal{M}}}_{{\rm{keep}}}={\rm{\varnothing }}$.
2. Set *i* = 0, *T*_tot_ = 0.
3. Initialize a pseudorandom generator with an *r*-bit seed *K*_seed_.
4. While $| {{\mathcal{M}}}_{{\rm{keep}}}|  < M$, run the following steps:
  - Challenge circuit generation subroutine: the client generates each of the circuits ${\{{C}_{i\cdot 2b+k}\}}_{k=1}^{2b}$ as follows.
    i. Initialize an empty circuit on *n* qubits.
    ii. For *j* = 1, …, *d*, run the following steps:
      A. Sample *n* SU(2) gates using the seeded pseudorandom generator and apply them to all *n* qubits.
      B. Apply the two-qubit gates corresponding to layer *T*_*j*_ of the chosen edge-coloured circuit topology.
    iii. Sample *n* SU(2) gates using the seeded pseudorandom generator and apply them to all *n* qubits.
  - Precheck: the client submits the precheck circuit *C*_precheck_ and waits for a response.
  - Client–server interaction subroutine:
    i. Start a timer.
    ii. The client submits the batch of circuits ${\{{C}_{i\cdot 2b+k}\}}_{k=1}^{2b}$ to the server.
    iii. The server responds with a batch of 2*b* bitstrings ${\{{x}_{i\cdot 2b+k}\}}_{k=1}^{2b}$.
    iv. Stop the timer. Record interaction time *T*_*b*_.
    v. Time out scenario: If *T*_b_ > *T*_*b*,cutoff_, then discard the batch.
    vi. If the batch is not discarded, then client computes ${{\mathcal{M}}}_{{\rm{keep}}}={{\mathcal{M}}}_{{\rm{keep}}}{\bigcup }_{k=1}^{2b}\{{x}_{i\cdot 2b+k}\}$ and accumulates the time *T*_tot_ = *T*_tot_ + *T*_b_.
    vii. Client increments the counter, *i* = *i* + 1.
5. Abort condition 1: if ${T}_{{\rm{tot}}}/| {{\mathcal{M}}}_{{\rm{keep}}}|  > {t}_{{\rm{threshold}}}$, then abort the protocol.
6. XEB score verification subroutine:
  - Test set construction: the client samples a subset ${\mathcal{V}}$ of size *m* randomly from ${{\mathcal{M}}}_{{\rm{keep}}}$ using the seeded pseudorandom generator.
  - Compute the score ${{\rm{XEB}}}_{{\rm{test}}}=\left(({2}^{n}/m)\cdot {\sum }_{j\in {\mathcal{V}}}| \langle {x}_{j}| {C}_{j}| 0\rangle {| }^{2}\right)-1$.
  - Abort condition 2: if XEB_test_ < *χ* then abort the protocol.
7. If not-abort, the client feeds the *M* samples *x*_1_, …, *x*_*M*_ together with the random seed *K*_ext_ to the extractor *Ext*.

Output: Conditioned on the protocol not aborting, the protocol returns Ext(*K*_ext_, (*x*_1_, …, *x*_*M*_)) as the final bitstring.

### Protocol security

Our primary theoretical contribution is the security of the implemented protocol against a restricted adversary. Our adversarial model considers realistic and near-term adversaries using best-known strategies (see Supplementary Information section IIIC for details). In brief, our adversary has a bounded classical computer and a quantum computer and uses both to generate the samples. Specifically, we make the following key assumptions about the adversary (further elaborated in Supplementary Information section IIIC):

1. The server does not perform any postselection attacks; that is, the *M* detected rounds in the protocol are a fair representation of the adversary behaviour.
2. Of the *M* valid samples, the server a priori selects *Q* rounds for which it honestly returns samples by executing the challenge circuit on the quantum computer. For the remaining *M* − *Q* samples, it returns deterministic samples obtained by simulating the circuits on a powerful classical computer (of power ${\mathcal{A}}$, measured in terms of number of floating point operations per second).
3. For each of the *Q* quantum rounds, it interacts only with the quantum computer once (it does not attempt to oversample a circuit).

In practice, these assumptions are probably stronger than necessary; we leave adaptation of the formal cryptographic protocol for a relaxed set of assumptions for future work.

To prove the security of the protocol, we prove a lower bound to the smooth min-entropy ${H}_{\min }^{{\varepsilon }_{{\rm{s}}}}({X}^{M}| {\widetilde{I}}_{{\rm{sn}}})$ of the bits before the extractor given this adversary, where ${\widetilde{I}}_{{\rm{sn}}}$ is the initial snapshot of side information minus the randomness extractor seed. To do so, we first provide a bound on the probability that the server executing a fixed number *Q* of quantum rounds passes the XEB test with threshold *χ* (see Supplementary Information section  IIID). We denote the event in which the protocol does not abort as *Ω*, the probability of not aborting as Pr[*Ω*] and the upper bound on the probability as *ε*_adv_(*Q*, *χ*).

Now, given a target not-abort probability *ε*_accept_ = 4*ε*_s_ (for an *ε*_sou_-sound protocol, *ε*_accept_ = 4*ε*_s_ = *ε*_sou_), the upper bound to Pr[*Ω*] allows us to compute *Q*_min_ = min{*Q*: *ε*_adv_(*Q*, *χ*) ≥ 4*ε*_s_}, which represents the minimum number of quantum rounds that the server needs to perform for the protocol to not abort with probability 4*ε*_s_. Given *Q*_min_, we bound the smooth min-entropy of the samples *X*^*M*^ given classical side information ${\widetilde{I}}_{{\rm{sn}}}$ using the following theorem.

#### Theorem 1

Let *Ω* denote the event in which the randomness certification protocol in Supplementary Information section IA does not abort and let *σ* be the state over registers *X*^*M*^ and ${\widetilde{I}}_{{\rm{sn}}}$. Given *ε*_s_ ∈ (0, 1/4), the protocol either aborts with a probability greater than 1 − 4*ε*_s_ or

4 $${H}_{\min }^{{\varepsilon }_{{\rm{s}}}}({X}^{M}| {\widetilde{I}}_{{\rm{sn}}})\ge {Q}_{\min }(n-1)+\log {\varepsilon }_{{\rm{s}}},$$

where ${Q}_{\min }=\arg \,\mathop{\min }\limits_{Q}\{{\varepsilon }_{{\rm{adv}}}(Q,\chi )\ge 4{\varepsilon }_{{\rm{s}}}\}$ and *ε*_adv_(*Q*, *χ*) is the upper bound to Pr(*Ω*).

## Online content

Any methods, additional references, Nature Portfolio reporting summaries, source data, extended data, supplementary information, acknowledgements, peer review information; details of author contributions and competing interests; and statements of data and code availability are available at 10.1038/s41586-025-08737-1.

## Supplementary information

Supplementary Information Supplementary Information sections 1–6, including Supplementary Figs. 1–7, Supplementary Tables 1–4 and Supplementary References; see Contents for details.

## Data Availability

The full data presented in this work are available at Zenodo (10.5281/zenodo.12952178).

## Code availability

The code required to verify and reproduce the results presented in this work is available at Zenodo (10.5281/zenodo.12952178).
