# Supplementary material for: Certified randomness using a trapped-ion quantum processor
Source: Nature. 2025 Mar 26;640(8058):343–8. doi: 10.1038/s41586-025-08737-1 (PMC11981928; doi:10.1038/s41586-025-08737-1)
Supplement: Supplementary file 1 — Supplementary Information sections 1–6, including Supplementary Figs. 1–7, Supplementary Tables 1–4 and Supplementary References; see Contents for details. [file 41586_2025_8737_MOESM1_ESM.pdf]

nature portfolio

https://doi.org/10.1038/s41586-025-08737-1

Supplementary information

Certified randomness using a trapped-ion quantum processor

In the format provided by the authors and unedited

Supplemental material for: Certified Randomness Using a Trapped-Ion Quantum Processor

Contents

I. Motivation: Complexity-Theoretic Security 2
A. Proof of Theorem 6 3
B. Multi-round Analysis 9
C. Limitations of Asymptotic Guarantees 9

II. Overview of the Protocol 9
A. Protocol Details 10
B. Necessary Conditions for Protocol Success 10
C. Difference from Existing Protocols 10

III. Security Analysis of the Protocol 11
A. Security Definition 11
B. XEB Preliminaries 12
1. Distribution of the XEB score of uniformly sampled bitstrings 12
2. Distribution of the XEB score of bitstrings perfectly sampled from the quantum state 13
3. Distribution of the XEB score of bitstrings sampled from a mixture 13
4. Distribution of the XEB score of bitstrings obtained by finite-fidelity quantum sampling 13
C. Adversarial Model and Assumptions 14
1. Circuit sampling and hardness assumptions 14
2. Assumptions on computing devices 16
3. Assumption on frugal rejection sampling 17
4. Adversary strategy restriction 18
D. Bounds on the Entropy Certified by the Protocol 18
E. Proof of Protocol Soundness 21
F. Randomness Expansion 22

IV. Details of Protocol Implementation 23
A. Quantum Circuit Simulation by Tensor Network Contraction 23
1. Index slicing 23
2. Trade-off between compute and memory operations 24
3. Simulation algorithm in our experiment 24
4. Heterogeneous high-performance computing platforms of our experiment 25
B. Selection of Experimental Parameters 26
C. Selection of Challenge Circuits 26
D. Client-Server Interaction 27
E. Verification 27
F. Randomness Extraction 28

V. Details on Outlook for Future Experiments 28

VI. Table of Variables 29

References 29

I. Motivation: Complexity-Theoretic Security

A certified randomness expansion protocol must generate randomness that is secure against potential adversaries. The adversary receiving the quantum circuits could be using strategies that provide less entropy than the client desires (or no entropy at all). Ref. [1] proves that if a device generates outputs that pass the XEB test with high enough probability, it must generate $\Omega(n)$ bits of entropy. However, Ref. [1] considers XEB tests for k samples generated from a fixed circuit, whereas our setting considers XEB tests for k samples generated from k distinct circuits because we request one sample per circuit. In the remainder of this section we extend the security analysis of Ref. [1] to our setting. In some cases we reference theorems stated in the extended arXiv version of Ref. [1], which is Ref. [2]. The notation in this section aims to be consistent with [1, 2] and differs from that used in the sections below. Readers who are uninterested in complexity-theoretic motivations can skip to other sections without issue.

This argument builds on the LLHA (Long-List Hardness Assumption) conjecture, which was stated and proven for a random oracle model in Ref. [1]. To state the conjecture, we first define the following problem:

Definition 1 (Long List Quantum Supremacy Verification $\mathrm{LLQSV}(\mathcal{D})$, restated from Problem 2 of [1])**.** *We are given oracle access to $M = O(2^{3n})$ quantum circuits $C_1, \ldots, C_M$, each on n qubits, which are promised to be drawn independently from the distribution $\mathcal{U}$. We are also given oracle access to M strings $s_1, \ldots, s_M \in \{0,1\}^n$. The LLQSV($\mathcal{D}$) task is to distinguish the following two cases:*

1. ***No-Case**: Each s_i is sampled uniformly from $\{0,1\}^n$.*
2. ***Yes-Case**: Each s_i is sampled from p_{C_i}, the output distribution of C_i.*

We denote the yes-case condition as $\vec{s} \sim p_{\vec{C}}$ and the no-case condition as $\vec{s} \sim \mathcal{U}^M$.

The LLHA conjecture is defined with respect to the above problem and a parameter B:

Definition 2 (Long List Hardness Assumption $\mathrm{LLHA}_B(\mathcal{D})$, restated from Eq. 3 of [1] and Assumption 5.1 of [2])**.** *For an n-qubit quantum circuit distribution $\mathcal{D}$ and some parameter $B < n$, denoted as $\mathrm{LLHA}_B(\mathcal{D})$, it holds that*

$$\mathrm{LLQSV}(\mathcal{D}) \notin \mathsf{QCAMTIME}(2^B n^{O(1)})/q(2^B n^{O(1)}), \tag{I.1}$$

where q denotes quantum advice.

Here, QCAM (Quantum Classical Arthur–Merlin) is a class of problems that admit an Arthur-Merlin protocol with classical communication and a quantum verifier. $\mathsf{QCAMTIME}(T)/q(A)$ is the generalization of QCAM, where the verifier can use running time T and receives A bits of quantum advice that depend only on n. To justify this conjecture, Ref. [1] shows that LLHA holds in the random oracle model. This serves as evidence that LLHA might hold for random circuit sampling.

The LLHA conjecture can be used to show that any algorithm that achieves a high cross-entropy benchmark score with a sufficiently high probability must necessarily generate entropy. In particular, we can define what we mean by "achieving a high cross-entropy benchmarking score."

Definition 3 (Linear Cross-Entropy Benchmarking $\mathrm{LXEB}_{b,k}(\mathcal{D})$, restated from Problem 1 of [1])**.** *Let $\mathcal{D}$ be a probability distribution over n-qubit quantum circuits. The $\mathrm{LXEB}_{b,k}(\mathcal{D})$ problem is to perform the following task [3]: given $C \sim \mathcal{D}$, output a set of k distinct bitstrings $\{z_1, \ldots, z_k\}$ such that*

$$\sum_{i=1}^{k} p_C(z_i) \geq \frac{bk}{N}, \tag{I.2}$$

where $p_C(z_i)$ is the probability that the output of quantum circuit C is z_i, and $N = 2^n$.

In other words, the $\mathrm{LXEB}_{b,k}$ problem amounts to drawing samples that achieve a cross-entropy benchmarking score, over k samples of a *single* circuit C, higher than some threshold b. We remark that here we add the requirement that the bitstrings must be distinct to avoid the situation where an adversary repeats a single large probability bitstring to get a high score. The presence of entropy in such samples is guaranteed by the following theorem.

Theorem 4 ($\mathrm{LXEB}_{b,k}$ ensures von Neumann entropy, restated from Theorem 5.10 of [2])**.** *For integer n, assume that $\mathrm{LLHA}_B(\mathcal{D})$ holds for distribution $\mathcal{D}$ over circuits acting on n qubits. Then for any device which on input of a circuit $C \sim \mathcal{D}$ outputs a classical state Z over $\{0,1\}^{nk}$ (k bitstrings, each of length n) solving $\mathrm{LXEB}_{b,k}$ with probability q, it holds that*

$$H(Z|C) \geq \frac{B}{2}\left(\frac{bq-1}{b-1} - n^{-\omega(1)}\right). \tag{I.3}$$

While the theorem above guarantees von Neumann entropy $H(Z|C)$ in samples that pass the $\mathrm{LXEB}_{b,k}$ problem, note that the statistic we use in the main text is slightly different from the statistic in Definition 3. In particular, whereas $\mathrm{LXEB}_{b,k}$ is defined with respect to many samples corresponding to the same circuit, the statistic we use is defined over a set of circuits. We define the modified problem in the same style as $\mathrm{LXEB}_{b,k}$:

Definition 5 (Mixed Linear Cross-Entropy Benchmarking $\mathrm{MLXEB}_{b,k}(\mathcal{D})$)**.** *Let $\mathcal{D}$ be a probability distribution over quantum circuits on n qubits. Then the $\mathrm{MLXEB}_{b,k}(\mathcal{D})$ problem is as follows: given $\vec{C}$ with $|\vec{C}| = k$ and with each C_i drawn from $\mathcal{D}$, output samples $z_1, \ldots, z_k \in \{0,1\}^n$ such that*

$$\sum_{i=1}^{k} p_{C_i}(z_i) \geq \frac{bk}{N}. \tag{I.4}$$

Our main result for this section is a proof ensuring entropy for an algorithm that solves the $\mathrm{MLXEB}_{b,k}(\mathcal{D})$ problem.

Theorem 6 ($\mathrm{MLXEB}_{b,k}$ ensures von Neumann entropy.)**.** *For integer n, assume that $\mathrm{LLHA}_B(\mathcal{D})$ holds for distribution $\mathcal{D}$ over circuits acting on n qubits. Then for any device that on input of a set of k independently sampled circuits $\vec{C}$ with $\vec{C} \sim \mathcal{D}^k$ outputs a classical state Z over $\{0,1\}^{nk}$ (k bitstrings, each of length n) solving $\mathrm{MLXEB}_{b,k}$ with probability q, it holds that*

$$H(Z|\vec{C}) \geq \frac{B}{2}\left(\frac{bq-1}{b-1} - n^{-\omega(1)}\right). \tag{I.5}$$

Proof. The proof is given in Section I A. □

Complexity-theoretic evidence that a device solving the $\mathrm{MLXEB}_{b,k}$ problem must generate entropy gives justification that our protocol based on the statistics defined in Eq. II.1 can be used for randomness certification and expansion. For notational convenience, for the rest of the paper we use the term "cross-entropy benchmarking score" to refer to $\mathrm{MLXEB}_{b,k}$. Furthermore, our primary statistic, the XEB score (defined in Eq. II.1) is the same as Eq. I.4 in the definition of $\mathrm{MLXEB}_{b,k}$ up to a normalization factor and a constant offset.

A. Proof of Theorem 6

To give a complexity-theoretic guarantee of entropy generation, we follow Ref. [2] in proving an intermediate result showing that a polynomial time quantum algorithm that solves LXEB while having low von Neumann entropy can be used to solve LLQSV, thereby violating LLHA. We begin by stating the LXEB version of the result (Theorem 7) and clarifying the difference from the result in [2]. Then we state the MLXEB version that applies to our experiment (Theorem 8) and provide a series of lemmas that are needed to prove both. Finally, we provide proofs for both theorems.

Theorem 7 (Low-entropy algorithm solving LXEB also solves LLQSV, modified from Theorem 5.8 of [2])**.** *Consider a device $\mathcal{A}$ which runs in quantum polynomial time and satisfies the following condition:*

$$H(Z|C)_{\mathcal{A}} < \frac{B}{2}\left(\frac{bq-1-\varepsilon}{b-1}\right), \tag{I.6}$$

$$q = \Pr_{C\sim\mathcal{D}, \vec{z}\sim\mathcal{A}(C)}\left[\sum_i p_C(z_i) \geq \frac{bk}{N}\right], \tag{I.7}$$

where $\varepsilon = n^{-O(1)}$ and q is the probability of $\mathcal{A}$ passing $\mathrm{LXEB}_{b,k}$. If such an $\mathcal{A}$ exists, then there is a quantum-classical Arthur-Merlin protocol which on input of an $O(n)$-bit advice string, solves $\mathrm{LLQSV}_B(\mathcal{D})$ in time $2^B n^{O(1)}$. In other words, $\mathrm{LLQSV}_B(\mathcal{D}) \in \mathsf{QCAMTIME}(2^B n^{O(1)})/O(n)$.

This theorem is modified from [2] since the original theorem requires $\mathcal{A}$'s min-entropy to be bounded, whereas our theorem requires $\mathcal{A}$'s von Neumann entropy to be bounded. It turns out that the original theorem is not technically possible. To see this, note that the original proof provides a lower bound on the single round min-entropy, which scales linearly in n. This is impossible: one can honestly perform quantum sampling 99% of the time and return the zero bitstring 1% of the time, which is an algorithm with min-entropy $H_{\min} = -\log_2 0.01$ regardless of n and passes LXEB with high probability. We fix this by bounding the von Neumann entropy for the single-round analysis. The technical issue with the analysis in [1, 2] that leads to

this impossible conclusion is that the set used in the approximate counting protocol is ill-defined since it depends on random samples from the quantum algorithm, which Arthur and Merlin cannot agree on. Our analysis below is free from this issue.

If LLHA is true, Theorem 7 implies that the output of the algorithm that solves LXEB must contain entropy. However, our experimental protocol does not check if the algorithm solves $\mathrm{LXEB}_{b,k}$ since we request one sample per circuit. Therefore, we wish to prove an equivalent theorem for the MLXEB case.

Theorem 8 (Low-entropy algorithm solving MLXEB also solves LLQSV)**.** *Consider a device $\mathcal{A}$ which runs in quantum polynomial time and satisfies the following condition:*

$$H(Z|\vec{C}) < \frac{B}{2}\left(\frac{bq-1-\varepsilon}{b-1}\right), \tag{I.8}$$

$$q = \Pr_{\vec{C}\sim\mathcal{D}^k, \vec{z}\sim\mathcal{A}(\vec{C})}\left[\sum_i p_{C_i}(z_i) \geq \frac{bk}{N}\right], \tag{I.9}$$

where $\varepsilon = n^{-O(1)}$. If such an $\mathcal{A}$ exists, $\mathrm{LLQSV}_B(\mathcal{D}) \in \mathsf{QCAMTIME}(2^B n^{O(1)})/O(n)$.

We provide the analysis for the MLXEB case. Adaptation of the proofs to the LXEB case is straightforward, and we remark on the necessary changes at the end of this section. We first prove that an algorithm satisfying the requirements of Theorem 8 must output heavy hitters (outputs with probability greater than $\tau/2^{B/2}$ for some $\tau \in (0,1]$) often.

Lemma 9. *An algorithm satisfying the requirements of Theorem 8 must output heavy hitters with probability at least p satisfying $p > \frac{b(1-q)+\varepsilon}{b-1}$, where*

$$p(\tau) = \Pr_{\vec{C}\sim\mathcal{D}^k, \vec{z}\sim\mathcal{A}(\vec{C})}\left[\Pr\left[\mathcal{A}(\vec{C}) = \vec{z}\right] \geq \frac{\tau}{2^{B/2}}\right], \tag{I.10}$$

and $p \equiv p(1)$.

Proof. With Markov's inequality, Eq. I.8 implies

$$\Pr_{\vec{C}\sim\mathcal{D}^k, \vec{z}\sim\mathcal{A}(\vec{C})}\left[\log\frac{1}{\Pr\left[\mathcal{A}(\vec{C}) = \vec{z}\right]} \geq \frac{B}{2}\right] \leq \frac{H(Z|\vec{C})}{B/2} < \frac{bq-1-\varepsilon}{b-1} \implies p > 1 - \frac{bq-1-\varepsilon}{b-1} = \frac{b(1-q)+\varepsilon}{b-1}. \tag{I.11}$$

□

We now examine the expectation value of a random variable $Y_\tau(\vec{C}, \vec{s})$:

$$Y_\tau(\vec{C}, \vec{s}) := \Pr_{\vec{z}\sim\mathcal{A}(\vec{C})}\left[\Pr\left[\mathcal{A}(\vec{C}) = \vec{z}\right] \geq \frac{\tau}{2^{B/2}} \wedge \exists i : z_i = s_i\right]. \tag{I.12}$$

We see that Y_τ is the probability that the output of $\mathcal{A}$ consists of heavy hitters with probability satisfying the conditions of Lemma 9, with at least one matching bitstring. Define its expectation value in the yes-case and no-case of LLQSV defined in Def. 1 as μ_1 and μ_0:

$$\mu_1(\tau) = \mathop{\mathbb{E}}_{\vec{C}\sim\mathcal{D}^k, \vec{s}\sim p_{\vec{C}}}[Y_\tau(\vec{C}, \vec{s})], \quad \mu_0(\tau) = \mathop{\mathbb{E}}_{\vec{C}\sim\mathcal{D}^k, \vec{s}\sim\mathcal{U}^k}[Y_\tau(\vec{C}, \vec{s})]. \tag{I.13}$$

The gap between μ_1 and μ_2 helps distinguish between the yes- and no-cases. Showing the gap between μ_1 and μ_0 requires the following lemma.

Lemma 10. *Denoting the condition that the output $\vec{z}$ passes $\mathrm{MLXEB}_{b,k}$ as $V(\vec{C}, \vec{z})$,*

$$\Pr_{\vec{s}\sim p_{\vec{C}}}\left[\exists i, z_i = s_i \middle| V(\vec{C}, \vec{z})\right] \geq \frac{bk}{N} - O\left(\frac{1}{N^2}\right). \tag{I.14}$$

Proof. Given any $\vec{z}$, whether $z_i = s_i$ is independent for different i since the s_i are independently sampled. Using the inclusion-exclusion principle to the second order,

$$\Pr_{\vec{s}\sim p_{\vec{C}}}[\exists i, z_i = s_i] \geq \sum_i \Pr[z_i = s_i] - \sum_{i<j} \Pr[z_i = s_i] \Pr[z_j = s_j] \tag{I.15}$$

$$\geq \sum_i \Pr[z_i = s_i] - \sum_i \Pr[z_i = s_i] \sum_i \Pr[z_i = s_i] \tag{I.16}$$

$$= \sum_i \Pr[z_i = s_i] - \left(\sum_i \Pr[z_i = s_i]\right)^2 \tag{I.17}$$

$$= \sum_i p_{C_i}(z_i) \left(1 - \sum_i p_{C_i}(z_i)\right). \tag{I.18}$$

The value of Eq. I.18 increases until $\sum_i p_{C_i} = 1/2$. For $\sum_i p_{C_i} > 1/2$, $\Pr[\exists i, z_i = s_i] > \frac{1}{2k} > bk/N$ since $k = O(n^2)$. Conditioned on passing $\mathrm{MLXEB}_{b,k}$, we have $\sum_i p_{C_i}(z_i) \geq bk/N$. Therefore,

$$\Pr_{\vec{s}\sim p_{\vec{C}}}\left[\exists i, z_i = s_i \middle| V(\vec{C}, \vec{z})\right] \geq \frac{bk}{N} - \frac{b^2k^2}{N^2}. \tag{I.19}$$

□

Now we show the gap.

Lemma 11. *For $\tau \in [0, 1]$,*

$$\frac{\mu_1(\tau)}{\mu_0(\tau)} \geq b \cdot \frac{p(\tau) + q - 1}{p(\tau)}. \tag{I.20}$$

Proof. For the yes-case,

$$\mu_1(\tau) = \mathop{\mathbb{E}}_{\vec{C}\sim\mathcal{D}^k, \vec{s}\sim p_{\vec{C}}}[Y_\tau(\vec{C}, \vec{s})] = \Pr_{\vec{C}\sim\mathcal{D}^k, \vec{s}\sim p_{\vec{C}}, \vec{z}\sim\mathcal{A}(\vec{C})}\left[\Pr\left[\mathcal{A}(\vec{C}) = \vec{z}\right] \geq \frac{\tau}{2^{B/2}} \wedge \exists i : z_i = s_i\right] \tag{I.21}$$

$$\geq \Pr_{\vec{C}\sim\mathcal{D}^k, \vec{s}\sim p_{\vec{C}}, \vec{z}\sim\mathcal{A}(\vec{C})}\left[\Pr\left[\mathcal{A}(\vec{C}) = \vec{z}\right] \geq \frac{\tau}{2^{B/2}} \wedge \exists i : z_i = s_i \wedge V(\vec{C}, \vec{z})\right] \tag{I.22}$$

$$= \Pr_{\vec{s}\sim p_{\vec{C}}}\left[\exists i : z_i = s_i \middle| \Pr\left[\mathcal{A}(\vec{C}) = \vec{z}\right] \geq \frac{\tau}{2^{B/2}} \wedge V(\vec{C}, \vec{z})\right] \Pr_{\vec{C}\sim\mathcal{D}^k, \vec{z}\sim\mathcal{A}(\vec{C})}\left[\Pr\left[\mathcal{A}(\vec{C}) = \vec{z}\right] \geq \frac{\tau}{2^{B/2}} \wedge V(\vec{C}, \vec{z})\right] \tag{I.23}$$

$$\geq \left(\frac{bk}{N} - O\left(\frac{1}{N^2}\right)\right) \cdot \Pr_{\vec{C}\sim\mathcal{D}^k, \vec{z}\sim\mathcal{A}(\vec{C})}\left[\Pr\left[\mathcal{A}(\vec{C}) = \vec{z}\right] \geq \frac{\tau}{2^{B/2}} \wedge V(\vec{C}, \vec{z})\right] \tag{I.24}$$

$$\geq \frac{bk}{N} \cdot (p(\tau) + q - 1) - O\left(\frac{1}{N^2}\right). \tag{I.25}$$

The fourth line holds by Lemma 10, and the last line holds by union bound.

For the no-case,

$$\mu_0(\tau) = \mathop{\mathbb{E}}_{\vec{C}\sim\mathcal{D}^k, \vec{s}\sim\mathcal{U}^k}[Y_\tau(\vec{C}, \vec{s})] = \Pr_{\vec{C}\sim\mathcal{D}^k, \vec{s}\sim\mathcal{U}^k, \vec{z}\sim\mathcal{A}(\vec{C})}\left[\Pr\left[\mathcal{A}(\vec{C}) = \vec{z}\right] \geq \frac{\tau}{2^{B/2}} \wedge \exists i : z_i = s_i\right] = \frac{k}{N} \cdot p(\tau). \tag{I.26}$$

By Eq. I.25 and I.26, we conclude the proof. □

Lemmas 9 and 11 of this work and Lemma 5.4 of [2] imply that for μ_1, μ_0 defined for an algorithm satisfying the requirement of Theorem 8,

$$\frac{\mu_1(\tau)}{\mu_0(\tau)} \geq \frac{\mu_1(1)}{\mu_0(1)} \geq b \cdot \frac{p + q - 1}{p} \geq 1 + \varepsilon. \tag{I.27}$$

Further, by the same argument used in Lemma 5.5 of [2] (modulo some typos), we have the following result. This result is necessary due to the need for Arthur to verify Merlin's claim in the Goldwasser-Sipser protocol. The manner in which this arises in our work can be seen in Eq. I.42. For Ref. [2], it is discussed before Lemma 5.5 and can be seen in Eq. 112. For both cases, it is due to the fact that Arthur cannot calculate some fraction with infinite precision.

Lemma 12. *For $T \geq \frac{16}{\varepsilon}\log\left(\frac{N}{\varepsilon}\right)$, there exists $j \in [T/2]$ such that*

$$\mu_1\left(1/2 + j/T\right) \geq (1+\varepsilon/2)\,\mu_0\left(1/2 + (j-1)/T\right). \tag{I.28}$$

Proof. We modify the proof as follows. First, $j \in [T/2]$, and for the product, j goes from 0 to $T/2-1$. Second, $\frac{8}{\varepsilon}\log\frac{n}{\varepsilon}$ should be $\frac{16}{\varepsilon}\log\frac{N}{\varepsilon}$. □

With this gap, a quantum-classical Arthur–Merlin protocol can be used to distinguish between the two cases. This is accomplished by first defining a set whose size depends on $\mathbb{E}[Y_\tau(\vec{C},\vec{s})]$, such that there is a gap in the set size between the two cases. This allows us to use the Goldwasser–Sipser protocol for approximate counting [4] to differentiate between the two cases. To show that there exists a set with a gap in the size, we use the following lemma.

Lemma 13. *Let $\varepsilon \in [0,1], \delta, a_0, a_1 \in (0,1]$ be real numbers satisfying $a_1 = a_0 + \delta \leq (1+\varepsilon/8)a_0$. Let X_0, X_1 be random variables in $[0,1]$ such that $X_b > 0$ implies $X_b \geq a_b$ for $b \in \{0,1\}$ and $\mathbb{E}[X_1] \geq (1+\varepsilon)\,\mathbb{E}[X_0]$. Then there exists a rational number $1 \geq t \geq a_0$ such that*

$$\Pr[X_1 > t + \delta/2] \geq (1+\varepsilon/4)\left(\Pr[X_0 > t] + \frac{\varepsilon}{8}\,\mathbb{E}[X_1]\right). \tag{I.29}$$

Proof. For $b \in \{0,1\}$, let f_b and F_b be the PDF and the CDF of X_b, respectively. Recall that by integration by parts,

$$\mathbb{E}[X_b] = \int_{a_b}^1 x f_b(x)\mathrm{d}x = xF_b(x)|_{a_b}^1 - \int_{a_b}^1 F_b(x)\mathrm{d}x = 1 - a_b\Pr[X_b \leq a_b] - \int_{a_b}^1 F_b(x)\mathrm{d}x \tag{I.30}$$

$$= a_b \Pr[X_b > a_b] + \int_{a_b}^1 \Pr[X_b > x]\mathrm{d}x. \tag{I.31}$$

The condition that $\mathbb{E}[X_1] \geq (1+\varepsilon)\,\mathbb{E}[X_0]$ implies that for $\varepsilon \leq 1$,

$$\mathbb{E}[X_1] \geq \left(1-\frac{\varepsilon}{4}\right)(1+\varepsilon)\,\mathbb{E}[X_0] + \frac{\varepsilon}{4}\cdot\mathbb{E}[X_1] \geq \left(1+\frac{\varepsilon}{2}\right)\mathbb{E}[X_0] + \frac{\varepsilon}{4}\cdot\mathbb{E}[X_1] \tag{I.32}$$

$$\geq \left(1+\frac{\varepsilon}{2}\right)\left(\mathbb{E}[X_0] + \frac{\varepsilon}{8}\cdot\mathbb{E}[X_1]\right). \tag{I.33}$$

This further implies one of the following two cases:

$$a_1\Pr[X_1 > a_1] \geq a_0\left(1+\frac{\varepsilon}{2}\right)\left(\Pr[X_0 > a_0] + \frac{\varepsilon}{8}\cdot\mathbb{E}[X_1]\right), \tag{I.34}$$

$$\int_{a_1}^1 \Pr[X_1 > x]\mathrm{d}x \geq (1+\varepsilon/2)\int_{a_0}^1\left(\Pr[X_0 > x] + \frac{\varepsilon}{8}\cdot\mathbb{E}[X_1]\right)\mathrm{d}x. \tag{I.35}$$

In the former case of Eq. I.34, since $a_1 \leq (1+\varepsilon/8)a_0$,

$$\Pr[X_1 > a_1] \geq \frac{1+\varepsilon/2}{1+\varepsilon/8}\left(\Pr[X_0 > a_0] + \frac{\varepsilon}{8}\,\mathbb{E}[X_1]\right) \tag{I.36}$$

$$\geq (1+\varepsilon/4)\left(\Pr[X_0 > a_0] + \frac{\varepsilon}{8}\,\mathbb{E}[X_1]\right). \tag{I.37}$$

In the latter case of Eq. I.35, we partition the interval $(a_0, 1]$ into $m = 2/\delta$ subintervals of equal length; that is, let $\alpha_\ell = a_0 + \delta\ell/2$ (hence $\alpha_0 = a_0$, $\alpha_2 = a_1$ and $\alpha_m = 1$) be the grid points and the subintervals be $\{[\alpha_{\ell-1}, \alpha_\ell] : \ell \in [m]\}$. Also define

$$A_\ell := \int_{\alpha_{\ell-1}}^{\alpha_\ell} \Pr[X_1 > x]\mathrm{d}x, \quad B_\ell := \int_{\alpha_{\ell-1}}^{\alpha_\ell}\left(\Pr[X_0 > x] + \frac{\varepsilon}{8}\cdot\mathbb{E}[X_1]\right)\mathrm{d}x. \tag{I.38}$$

Now by Eq. I.35, we have

$$\sum_{\ell=3}^m A_\ell \geq (1+\varepsilon/2)\sum_{\ell=1}^m B_\ell \geq (1+\varepsilon/2)\sum_{\ell=1}^{m-2} B_\ell. \tag{I.39}$$

This implies that there exists $\ell \in [m-2]$ such that $A_{\ell+2} \geq (1+\varepsilon/2)B_\ell$. Since $\Pr[X > x]$ is monotonically nonincreasing, we have $\Pr[X_1 > \alpha_{\ell+1}] \geq A_{\ell+2}$ and $B_\ell \geq \Pr[X_0 > \alpha_\ell]$, and therefore

$$\Pr[X_1 > t + \delta/2] \geq (1+\varepsilon/2)\left(\Pr[X_0 > t] + \frac{\varepsilon}{8}\,\mathbb{E}[X_1]\right) \tag{I.40}$$

for $t = \alpha_\ell = a_0 + \ell\delta/2$. □

We now apply this lemma to our setting. Define $Z_{\tau,t}(\vec{C},\vec{s}) := \mathbb{1}[Y_\tau(\vec{C},\vec{s}) > t]$.

Corollary 14. *Assume that there is $1 \geq \varepsilon = n^{-O(1)}$ such that for $T \geq \frac{16}{\varepsilon}\log(\frac{N}{\varepsilon}))$ there is $\tau \in [1/2, 1]$ such that the inequality (I.28) holds. Then for $\delta = \frac{1}{2^{B/2}T}$, there exists $t \geq \frac{1}{2^{B/2}}(\tau - 1/T)$ such that*

1. $\nu_1 = \Pr_{\vec{C}\sim D^k, \vec{s}\sim p_{\vec{C}}} \left[Z_{\tau,t+\delta/2}\right] \geq \Omega(\varepsilon^2 k/N)$*, and*

2. $\nu_0 = \Pr_{\vec{C}\sim D^k, \vec{s}\sim U^k} [Z_{\tau-1/T,t}] \leq (1+\varepsilon/8)^{-1}\nu_1$.

Proof. We choose X_1 to be $Y_\tau(\vec{C},\vec{s})$ for $\vec{s} \sim p_{\vec{C}}$ and $\vec{C} \sim \mathcal{D}^k$. Similarly, we choose X_0 to be $Y_{\tau-1/T}(\vec{C},\vec{s})$ for $\vec{C} \sim \mathcal{D}^k$ and $\vec{s} \sim \mathcal{U}^k$. Then, by Eq. I.28, we have $\mathbb{E}[X_1] \geq (1+\varepsilon/2)\,\mathbb{E}[X_0]$.

By definition, $Y_\tau(\vec{C},\vec{s}) > 0$ implies that there exists a $\vec{z}$ that is output by $\mathcal{A}(\vec{C})$ with probability $\tau/2^{B/2}$ and there is an index $i \in [k]$ such that $s_i = z_i$. The probability of sampling such a $\vec{z}$ is at least $\frac{\tau}{2^{B/2}}$. Thus $Y_\tau(\vec{C},\vec{s}) > 0$ implies that $Y_\tau(C,s) > \frac{\tau}{2^{B/2}}$. To apply Lemma 13, we set $a_0 = \frac{\tau-1/T}{2^{B/2}}$, $\delta = \frac{1}{T2^{B/2}}$, and $a_1 = a_0 + \delta$. The ratio $\frac{a_1}{a_0} = \frac{\tau}{\tau-1/T} \leq 1 + \frac{2}{\tau T} = 1 + o(\varepsilon)$ for $T \geq \Omega(\frac{1}{\varepsilon}\log(N/\varepsilon))$. Now applying Lemma 13, there exists t such that

$$\Pr_{\vec{C},\vec{s}\sim p_{\vec{C}}}[Y_\tau(\vec{C},\vec{s}) > t+\delta/2] \geq \left(1+\frac{\varepsilon}{8}\right)\left(\Pr_{\vec{C}\sim\mathcal{D}^k,\vec{s}\sim U^k}[Y_{\tau-1/T}(\vec{C},\vec{s}) > t] + \frac{\varepsilon}{16}\mathop{\mathbb{E}}_{\vec{C}\sim\mathcal{D}^k,\vec{s}\sim p_{\vec{C}}}[Y_\tau(\vec{C},\vec{s})]\right). \tag{I.41}$$

This immediately implies the second conclusion. Further, since $\mathbb{E}_{\vec{C}\sim\mathcal{D}^k,\vec{s}\sim p_{\vec{C}}}[Y_\tau(\vec{C},\vec{s})] \geq bk(p(\tau)+q-1)/N \geq \varepsilon k/N$, plugging it into Eq. I.41 implies the first conclusion. □

The first conclusion in Corollary 14 implies that the event of acceptance cannot occur with zero probability, and thus there is always a suitable choice of the range R of the hash function in the QCAM protocol. The second implies that for a sufficiently long list of size $M \geq N^3$, the number of 1's in the yes case must be at least $(1+O(\varepsilon))$ times that in the no case with overwhelming probability. We will formally show these in the following proofs.

We now explain how to approximately compute $Z_{\tau,t}(\vec{C},\vec{s})$ in quantum time $2^B n^{O(1)}$ given $\vec{C},\vec{s}$: Consider the following sampling process $\mathcal{Z}(\vec{C},\vec{s})$ to confidence level $1-\eta$ for $\eta = 2^{-n^{O(1)}}$:

1. For $\delta := \frac{1}{2^{B/2}T}$, take $K = \frac{2}{\delta^2}((nk+2)\ln 2 + \ln(1/\eta)) = 2^B n^{O(1)}$ samples $\vec{z}_1,\ldots,\vec{z}_K \sim \mathcal{A}(\vec{C})$ to obtain an approximation of $\tilde{p}_{\mathcal{A}(\vec{C})}$ of the distribution of $\mathcal{A}(\vec{C})$.

2. Take $L = \frac{32}{\delta^2}\ln(2/\eta) = 2^B n^{O(1)}$ samples $\vec{z}_1,\ldots,\vec{z}_L \sim \mathcal{A}(\vec{C})$, and count the fraction of samples

$$\tilde{y} = \frac{1}{L}\cdot\#\left\{j \in [L] : \tilde{p}_{\mathcal{A}(\vec{C})}(\vec{z}_j) \geq \frac{\tau-1/2T}{2^{B/2}} \wedge \exists i : z_{j,i} = s_i\right\}, \tag{I.42}$$

where $z_{j,i}$ is the ith entry of $\vec{z}_j$.

3. If $\tilde{y} \geq t + \delta/4$, output 1; otherwise output 0.

To prove that the sampling process $\mathcal{Z}$ produces the correct output with high probability (Lemma 16), we prove that each step of $\mathcal{Z}(\vec{C},\vec{s})$ has low errors.

Lemma 15. *Step 1 outputs an approximation $\tilde{p}_{\mathcal{A}(\vec{C})}$ that is close to $\mathcal{A}(\vec{C})$'s distribution $p_{\mathcal{A}(\vec{C})}(\vec{z}) = \Pr[\mathcal{A}(C) = \vec{z}]$ in ℓ_∞-distance at most $\delta/2$ with probability at least $1-\eta/2$.*

Proof. By Hoeffding's inequality, for every $\vec{z} \in \{0,1\}^{nk}$, with probability at least $1 - \eta/2^{nk+1}$,

$$\left|p_{\mathcal{A}(\vec{C})}(\vec{z}) - \tilde{p}_{\mathcal{A}(\vec{C})}(\vec{z})\right| \leq \frac{\delta}{2}. \tag{I.43}$$

By a union bound, with probability at most $\eta/2$, every O satisfies (I.43), implying that their ℓ_∞-distance is at most $\delta/2$. □

Lemma 16. *The sampling process $\mathcal{Z}$ solves the promise problem of distinguishing (yes) $Z_{\tau,t+\delta/2}(\vec{C},\vec{s}) = 1$ and (no) $Z_{\tau-1/T,t}(\vec{C},\vec{s}) = 0$ to within error η. That is,*

1. *If* $Z_{\tau,t+\delta/2}(\vec{C},\vec{s})=1$*, then* $\mathcal{Z}(\vec{C},\vec{s})=1$ *with probability at least* $1-\eta$*.*

2. *If* $Z_{\tau-1/T,t}(\vec{C},\vec{s})=0$*, then* $\mathcal{Z}(\vec{C},\vec{s})=0$ *with probability at least* $1-\eta$*.*

Furthermore, $\mathcal{Z}$ *runs in time* $2^B n^{O(1)}$ *for* $\eta = 2^{-n^{O(1)}}$*.*

Proof. For $\vec{C},\vec{s}$ satisfying $Z_{\tau,t+\delta/2}(\vec{C},\vec{s})=1$, by Hoeffding's inequality, with probability at least $1-e^{-L\delta^2/32}=1-\eta/2$,

$$\tilde{y} \geq \Pr_{\vec{z}\sim\mathcal{A}(\vec{C})}\left[\tilde{p}_{\mathcal{A}(\vec{C})}(\vec{z}) \geq \frac{\tau}{2^{B/2}} - \frac{\delta}{2} \wedge \exists i : z_i = s_i\right] - \frac{\delta}{8}. \tag{I.44}$$

Then, assume that the approximation $\tilde{p}_{\mathcal{A}(\vec{C})}(\vec{z})$ obtained from Step 1 is close to $p_{\mathcal{A}(\vec{C})}(\vec{z})$ in ℓ_∞-distance $\delta/2$, which happens with probability at least $1-\eta/2$ by Lemma 15,

$$\tilde{y} \geq \Pr_{\vec{z}\sim\mathcal{A}(\vec{C})}\left[\Pr\left[\mathcal{A}(\vec{C})=\vec{z}\right] \geq \frac{\tau}{2^{B/2}} \wedge \exists i : z_i = s_i\right] - \frac{\delta}{8} = Y_\tau(\vec{C},\vec{s}) - \frac{\delta}{8} > t + \frac{3\delta}{8}. \tag{I.45}$$

The last inequality follows from the assumption that $Y_\tau(\vec{C},\vec{s}) > t+\delta/2$.

For $(\vec{C},\vec{s})$ satisfying $Z_{\tau,t}(\vec{C},\vec{s})=0$, again by Hoeffding's inequality, with probability at least $1-\eta/2$,

$$\tilde{y} \leq \Pr_{\vec{z}\sim\mathcal{A}(\vec{C})}\left[\tilde{p}_{\mathcal{A}(\vec{C})}(\vec{z}) \geq \frac{\tau}{2^{B/2}} - \frac{\delta}{2} \wedge \exists i : z_i = s_i\right] + \frac{\delta}{8}. \tag{I.46}$$

Again, with probability at least $1-\eta/2$, $\tilde{p}_{\mathcal{A}(\vec{C})}(\vec{z})$ is $\delta/2$ close to $p_{\mathcal{A}(\vec{C})}(\vec{z})$,

$$\tilde{y} \leq \Pr_{\vec{z}\sim\mathcal{A}(\vec{C})}\left[\Pr\left[\mathcal{A}(\vec{C})=\vec{z}\right] \geq \frac{\tau}{2^{B/2}} - \delta \wedge \exists i : z_i = s_i\right] + \frac{\delta}{8} = Y_{\tau-1/T}(\vec{C},\vec{s}) + \frac{\delta}{8} \leq t + \frac{\delta}{8}. \tag{I.47}$$

The last inequality follows from the assumption that $Y_{\tau-1/T}(\vec{C},\vec{s}) \leq t$. Combined with the observation that $\mathcal{Z}$ runs in time $2^B n^{O(1)}$ for $\eta = 2^{-n^{O(1)}}$ (since $K = 2^B n^{O(1)}$ samples are obtained by calling $\mathcal{A}(\vec{C})$ in step 1), this completes the proof. □

Now we describe the QCAM protocol as follows.

1. Both Arthur and Merlin are given access to classical advice τ, T, t, R (δ can be computed from them).

2. Arthur sends a hash function h that maps $[M] \to [R]$ and a random image $y \in [R]$.

3. Merlin sends an index i.

4. Arthur accepts if $h(i)=y$ and $\mathcal{Z}(\vec{C}_i,\vec{s}_i)=1$, and rejects otherwise.

Lemma 17. *For* $M' \geq N^3/k$ *size-*k *sets of circuits and bitstrings, there exist integers* $\kappa \in [M']$ *such that with probability at least* $1-e^{-\Omega(N\varepsilon^6)}$*, Arthur accepts between* κ *and* $(1+O(\varepsilon))\kappa$ *tuples in a yes instance and accepts at most* $\kappa' = (1-\varepsilon/32)\kappa$ *tuples in a no instance.*

Proof. In the yes-case, by Corollary 14, $\nu_1 = \Omega(\varepsilon^2 k/N)$. The probability that Arthur accepts $\mathcal{Z}(\vec{C},\vec{s})$ is at least $\nu_1 - \eta$ for $\eta = 2^{-n^{O(1)}}$ by Lemma 16. For $M' = N^3/k$, by Hoeffding's inequality, with probability at least $1-2e^{-M'(\nu_1-\eta)^2\xi^2} \geq 1-2e^{-\Omega(N\varepsilon^4 k\xi^2)}$, the number of accepted tuples is between $[(1-\xi)M'(\nu_1-\eta),(1+\xi)M'(\nu_1-\eta)]$ for some parameter $\xi \leq \varepsilon$ to be determined later. We may choose $\kappa = (1-\xi)M'(\nu_1-\eta)$.

In the no-case, the probability that Arthur accepts $\mathcal{Z}(\vec{C},\vec{s})$ is at most $\nu_0+\eta$ by Lemma 16. Let $\tilde{\nu}_0$ denote the fraction of tuples accepted by $Z_{\tau-1/T,\delta}$ in a no instance. By Hoeffding's inequality, for $\varepsilon \leq 1$,

$$\begin{aligned}
\Pr\left[\tilde{\nu}_0 \geq (1-\xi)^2(\nu_1-\eta)\right] &\leq \Pr[\tilde{\nu}_0 - (\nu_0+\eta) \geq (1-2\xi)(\nu_1-\eta) - (\nu_0+\eta)] && \text{(I.48)}\\
&\leq \Pr[\tilde{\nu}_0 - (\nu_0+\eta) \geq (1-2\xi)(\nu_1-\eta) - ((1-\varepsilon/8)\nu_1+\eta)] && \text{(I.49)}\\
&\leq e^{-M'(\varepsilon/8-2\xi)^2\nu_1^2 + O(M'\eta)} = e^{-\Omega(\varepsilon^6 kN)} && \text{(I.50)}
\end{aligned}$$

for $\xi = \varepsilon/32 \leq 1/32$. We may choose $\kappa' = (1-\xi^2)M'(\nu_1-\eta)$.

Therefore, the ratio is

$$\frac{\kappa'}{\kappa} \leq \frac{(1-\xi)^2(\nu_1-\eta)}{(1-\xi)(\nu_1-\eta)} = 1-\xi = 1-\varepsilon/32. \tag{I.51}$$

□

We are now ready to prove our main result.

Proof of Theorem 8. Given $M \geq N^3$ circuits and bitstrings, we can group them into $M' = (M/k)$ size-k sets of circuits and bitstrings. By Lemma 17 of this work and Lemma 5.2 of [2], for $R = 64\alpha\kappa/\varepsilon$, since $\alpha = 1 + O(\varepsilon)$, the gap is $\Omega(\varepsilon^2)$. Thus running an $(1/\varepsilon)^{O(1)}$-fold parallel repetition of the above protocol yields a constant gap. Since $1/\varepsilon = n^{O(1)}, T = n^{O(1)}, R \in [M]$, the advice τ, T, t, R all require relative precision at most $1/N^{O(1)}$, and hence the length is $O(n)$. □

Proof of Theorem 6. Assuming LLHA holds, Theorem 8 implies that the output of the algorithm that solves MLXEB must contain entropy, completing the proof of Theorem 6. □

Proof of Theorem 7. The proof for the LXEB case follows trivially by first changing $\vec{C}, \mathcal{D}^k, \vec{s}, \mathcal{U}^k$ into $C, \mathcal{D}, s, \mathcal{U}$ and the condition $\exists i : z_i = s_i$ into $s \in \vec{z}$. Furthermore, instead of Lemma 10, we use Eq. 103 of [2]. The rest of the analysis goes through unchanged. □

B. Multi-round Analysis

The single-round result of Theorem 4 (or Theorem 5.10 of [2]) and Theorem 6 does not result in a complete and sound single-round protocol. The first reason is that even for a device without entropy, the theorems only guarantee $bq < 1$. However, since b cannot be larger than 2 for an honest quantum computer to pass with overwhelming probability, we can only say a device without entropy must fail with probability greater than or equal to $1/2$. Instead, we need it to fail with overwhelming probability.

As such, a multi-round protocol is necessary, which is presented in Figure 1 of [2]. Theorem 5.11 of [2] shows that the multi-round protocol has a lower bound on the smooth min-entropy linear in the number of rounds and the number of qubits, and this result is obtained using the entropy accumulation theorem (EAT). We note that the EAT used here is a modified version in Section 4 of [2] instead of the original one in [5]. The application of EAT in [2] uses the single round von Neumann entropy bound provided by Theorem 5.10 of [2]. This is not affected by the fact that Corollary 5.9 of [2] is incorrect since Theorem 5.10 of [2] is still correct (use our Theorem 7 instead of Theorem 5.8 of [2]).

We can define a similar multi-round protocol, where the only difference is instead of sending one circuit per round, we send k circuits. In the same manner as [2], we should be able to apply EAT using the single round von Neumann entropy bound provided by Theorem 6, which allows us to obtain a lower bound on the smooth min-entropy for the modified multi-round protocol. We do not reproduce the multi-round analysis here.

C. Limitations of Asymptotic Guarantees

Three main challenges limit the applicability of the asymptotic result in Theorem 6 to our finite-sized experiment: our experiment is not in the asymptotic regime, the values of constants in Eq. I.5 are not known for our experiment, and there are differences between the protocol we implement experimentally and the protocol required by the asymptotic analysis.

First, the statement of Theorem 6 applies in the asymptotic limit of large n and extending it to finite-sized experiments would require further refinement of the analysis in Sec. I.

Second, the analysis hinges on the correctness of $\mathrm{LLHA}_B(\mathcal{D})$. Although an upper bound of $B \leq n/2$ is known due to Grover's algorithm, there is no lower bound on B for any distribution $\mathcal{D}$.

Third, the analysis guarantees single-round entropy for an output of k bitstrings. To lower bound the entropy across multiple rounds, Ref. [1] defines a protocol and proves entropy accumulation over multiple rounds, but the protocol is very different from our experiment. In particular, the authors perform multiple rounds of XEB tests to estimate the probability of passing the test, which allows them to lower bound the entropy. If each round is over hundreds of circuits and we have hundreds of test rounds, the cost of computing the probabilities would be prohibitive.

For these reasons, we cannot determine the soundness of our protocol solely on the basis of complexity-theoretic analysis that may or may not apply for experiments of our scale. Consequently, we instead focus on finite-sized adversaries performing realistic attacks with numerically bounded computational power.

II. Overview of the Protocol

To address the limitations of the asymptotic guarantees presented above, we implement a slightly modified protocol in our experiment, the security of which we analyze against a finite-sized adversary in Sec. III. The protocol has been presented in Methods and Fig. 1 of the main text, and we briefly summarise it below.

A. Protocol Details

The protocol consists of four main steps: (1) challenge circuit generation, (2) client-server interaction, (3) XEB score verification, and (4) randomness extraction.

1. **Challenge Circuit Generation**: The client generates a large number of n-qubit challenge circuits C_i using a pseudorandom number generator with a r-bit random seed. The circuits are chosen to balance the following two considerations: (1) no classical adversary should be able to simulate the circuits within the time that a quantum computer takes to run them, and (2) a classical supercomputer should be able to validate them. Detailed discussion on circuit selection is deferred to Sec. IV C. These circuits are kept secret from the client.

2. **Client-Server Interaction**: The client sends a batch of circuits to the quantum server and requests one sample from the output distribution of each circuit. The circuits are submitted in batches of b jobs, where each job consists of two circuits stitched via a layer of mid-circuit measurement and reset. Such "batching" and "stitching" allow us to amortize network latencies and execution overheads across $2b$ samples (Note: the symbol b used here to denote batch size is not related to the symbol b used to parameterize LLHA in I). Each batch is preceded by an agreed-upon "precheck" circuit, which announces the client's intention to submit a batch, checks for the server's readiness to accept jobs, and triggers calibrations if necessary. For each batch, the quantum server executes the circuits on the quantum computer and returns the samples $\{x_1, \ldots, x_{2b}\}$, with $x_i \in \{0,1\}^n$. On the Quantinuum H2-1 hardware, detectable faults such as loss of an ion from the trap may prevent a batch from completing execution. As a result, if the entire batch is not returned within a time of $T_{b,\text{cutoff}}$, all outstanding jobs are cancelled, and any samples collected from this batch are discarded. The client-server interaction continues until we receive M valid samples. If the average time per sample of the retained rounds exceeds a threshold $t_{\text{threshold}}$, the protocol aborts.

3. XEB **Score Verification**: Having collected M samples, $\{x_i\}_{i\in[M]}$, corresponding to M circuits, the client randomly selects a subset of circuit-bitstring pairs defined by a set of m indices $\mathcal{V} \subset [M]$ and computes the linear cross-entropy benchmarking score, referred to as XEB_{test} throughout, which we define as

$$\text{XEB}_{\text{test}} = \frac{N}{m}\sum_{i\in\mathcal{V}} p_{C_i}(x_i) - 1, \tag{II.1}$$

where $N = 2^n$ and $p_{C_i}(x_i)$ is the probability of measuring the bitstring x_i after executing circuit C_i on an ideal quantum computer. These probabilities are computed classically on a large supercomputer by simulating $\{C_i\}_{i\in\mathcal{V}}$. If XEB_{test} is smaller than the protocol parameter χ, the client aborts the protocol.

4. **Randomness Extraction**: If the protocol does not abort, the client uses a seeded randomness extractor with seed K_{ext} and input $X^M := \{x_1, \ldots, x_M\}$ to extract the final random bits in register K.

B. Necessary Conditions for Protocol Success

Let $\mathcal{A}$ denote the power of the adversary's supercomputer (e.g., measured by the number of floating-point operations per second, or FLOPS), and let $\mathcal{B}$ denote the cost to simulate a given circuit exactly (e.g., measured by the number of floating-point operations, or FLOP count). Let $t_{\text{threshold}}$ denote the average allowed time that the quantum computer can take to return one sample. Since the circuit simulation cost scales linearly in the target fidelity of simulation when using exact tensor network contraction, the adversary can simulate each circuit to a fidelity of $\mathcal{A}\cdot t_{\text{threshold}}/\mathcal{B}$ in time $t_{\text{threshold}}$. Then, the protocol is secure only if an honest quantum server's fidelity on challenge circuits, ϕ, is much larger than the fidelity a classical adversary may obtain. That is,

$$\phi \gg \mathcal{A}\cdot t_{\text{threshold}}/\mathcal{B}. \tag{II.2}$$

Loosely speaking, the protocol guarantees true entropy resulting from the measurement of a quantum state only if three conditions are met. First, the quantum computer must be able to execute the challenge circuits with a high fidelity ϕ. Second, the quantum computer must be able to return bitstrings within a short time $t_{\text{threshold}}$. Third, the verifier should be able to validate circuits with high simulation cost $\mathcal{B}$ using supercomputers. In our implementation, all three conditions are satisfied.

C. Difference from Existing Protocols

In the protocols presented and analyzed in Refs. [1, 6], many samples from the same circuit C are demanded in each round, and the cross-entropy benchmarking score is computed over those samples for each circuit. Specifically, in Refs. [1, 6] the pertinent

score is $\text{XEB} = N\langle p_{\text{C}}(x_i)\rangle_{i\in\mathcal{V}} - 1$. In contrast, in our protocol we demand only a single bitstring from each circuit, and we compute the XEB score as an average over multiple circuits C_i: $\text{XEB} = N\langle p_{C_i}(x_i)\rangle_{i\in\mathcal{V}} - 1$. This difference is motivated by the fact that, for an adversarial server that may be trying to pass off classically simulated samples as true measurements of a quantum circuit, classically sampling many shots of a single circuit is much easier than sampling a single shots for many circuits. In other words, classical simulation cost is strongly sublinear in the number of shots. For example, this is shown in Figure 2 of Ref. [7]. At the same time, for the trapped-ion quantum processor that we use in this experiment, the device runtime scales nearly linearly with the number of shots.

III. Security Analysis of the Protocol

Our experiment is motivated by complexity-theoretic security guarantees discussed in Sec. I. However, these guarantees do not directly apply to our finite-sized experiment (Sec. I C). In this section we analyze the protocol as implemented and provide a proof that it is secure against a restricted class of adversaries operating under reasonable assumptions.

We note that the adversary we consider here is chosen such that it implements state-of-the-art techniques on both classical and quantum computers that can be realized in the near term. Therefore, it can only execute a very limited set of actions, and the results in this section do not apply if the actual adversary strategy deviates from our prescription. We discuss the details of the adversary model itself and the limitations of the model in detail in Section III C. Nevertheless, the analysis provides insight into the relationship between security, entropy, quantum computer fidelity and speed, and classical computational power, and provides order-of-magnitude estimates relevant for near-term implementations.

When we determine the amount of entropy safe to extract, we must make an assumption on the capabilities of the adversary. A way to numerically estimate a confidence on security is to an assume unreasonably powerful adversary (say 10 or 100 times more powerful than expected). We do explicitly acknowledge that if the actual adversary is fundamentally different from the analyzed adversary (e.g. a fault tolerant quantum computer executing a novel low-entropy algorithm that scores high on XEB, or a completely novel classical technique that significantly outperforms current state-of-the-art), it is not sufficient to simply adjust the power of the adversary in our model and a fundamentally different approach would be needed.

A. Security Definition

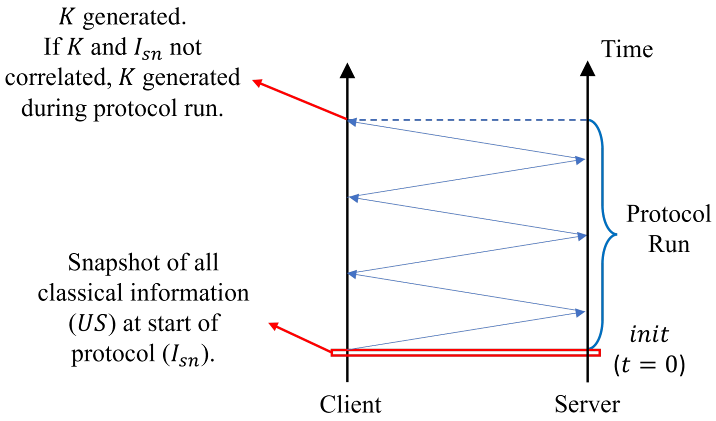

FIG. S1: Illustration of the security definition of certified randomness. In the ideal functionality, the generated bitstring K should be random and independent on any classical information available at the start of the protocol (described as a snapshot I_{sn} of all classical information).

In this section we formalize the definition of "security" for certified randomness. In the protocol, a classical client U interacts with a remote server $\tilde{S}$. Since we do not assume an authenticated communication channel between the client and the server, we have to account for other parties (denoted by E) who may have access to the communication channel. To simplify the analysis, we can encapsulate the remote server $\tilde{S}$ and other parties E into a single entity, which we refer to as the server $S = \tilde{S}E$ in the rest of the manuscript. If authenticated communication is present between the client and server, we refer to the remote server as the server instead, that is, $S = \tilde{S}$.

With this setup let us consider what it means to have "certified" randomness. The goal of "certified" randomness is to obtain a bitstring K that is "unpredictable" and that can be certified as such. For simplicity, we assume that there is no initial quantum

state in the server's quantum memory at the beginning of the protocol [1]. We leave a proper accounting of quantum memory to future work. Furthermore, we assume that any classical random variables used in the protocol have been generated and stored in their respective devices at this point [2] (e.g., extractor seed K_{ext}). Now, consider the state of the entire system US before the start of the protocol at an initial moment, and take a snapshot of the system (namely, a copy of all classical registers in the system) at the initial moment ($t = 0$), labeled as $I_{\text{sn}} = U^0 S^0$ (where S includes both server $\tilde{S}$ and environment E). We can then define the "unpredictability" of bitstring K as the property that K is uniformly random and uncorrelated to I_{sn}, i.e. that no classical information at the start of the protocol (captured in I_{sn}) can influence or predict the bitstring K, as shown in Fig. S1. Intuitively, if K and I_{sn} are uncorrelated, the source of randomness for K cannot be classical, since K could otherwise be deterministically computed from I_{sn}. This leads to the conclusion that the source of randomness in K must be quantum. As such, we can define the ideal functionality of the protocol as generating a bitstring K that is (1) uniformly distributed and (2) statistically independent of I_{sn} when the protocol does not abort. When the protocol aborts, no bitstring K is generated, and we label $K = \perp$. If Ω denotes the event that the ideal protocol does not abort (its complement Ω^c is the event that the protocol aborts), then the joint probability distribution of KI_{sn} after an ideal protocol, represented by a density matrix, is of the form

$$\rho^{\text{ideal}}_{KI_{\text{sn}}} = \tau_K \otimes \rho_{I_{\text{sn}}\wedge\Omega} + |\perp\rangle\langle\perp|_K \otimes \rho_{I_{\text{sn}}\wedge\Omega^c}, \tag{III.1}$$

where τ_K is the maximally mixed state representing a uniform distribution of strings of length $|K|$. In the above, we used the notation $\rho_{XA\wedge\Omega} = \sum_{x\in\Omega} p(x)\, |x\rangle\langle x| \otimes \rho^x_A$ for the subnormalized conditional states of the classical-quantum state $\rho_{XA} = \sum_x p(x)\, |x\rangle\langle x| \otimes \rho^x_A$ conditioned on the event Ω. For simplicity, we label $\wedge\Omega$ only at the end, e.g. we label $(\tau_K \otimes \rho_{I_{sn}})_{\wedge\Omega}$ as $\tau_K \otimes \rho_{I_{sn}\wedge\Omega}$.

In practice, the output K of a protocol is close to the ideal state in Eq. (III.1) only up to some security parameter. We refer to this distance as the soundness or the security parameter. In Eq. (III.1), an "abort" event always leads to $K = \perp$. Therefore, it suffices to consider the distance from ideal in the event the protocol does not abort. We can formally state the security definition as follows.

Definition 1 (Soundness of certified randomness protocol)**.** *Let $\varepsilon_{\text{sou}} \in (0, 1]$. A randomness certification protocol is ε_{sou}-sound if for an honest classical client and any server,*

$$\|\rho_{KI_{\text{sn}}\wedge\Omega} - \tau_K \otimes \rho_{I_{\text{sn}}\wedge\Omega}\|_{\text{Tr}} \leq \varepsilon_{\text{sou}}, \tag{III.2}$$

where $\|\cdot\|_{\text{Tr}}$ is the trace distance measure.

B. XEB Preliminaries

In our protocol, the client estimates the amount of entropy received by measuring the XEB score [8] over a set $\mathcal{V}$ of $m = |\mathcal{V}|$ challenge circuits:

$$\text{XEB}_{\text{test}} = \frac{N}{m} \sum_{i\in\mathcal{V}\subset[M]} p_{C_i}(x_i) - 1, \tag{III.3}$$

where $N = 2^n$ and $p_{C_i}(x_i)$ is the probability amplitude $|\langle x_i|C_i|0\rangle|^2$. At a high level, if the samples x_i are uniform, the XEB score is expected to be zero. On the other hand, if the server is using a perfect quantum computer to honestly sample from the corresponding quantum state, the XEB score is expected to be one. Similarly, a finite-fidelity quantum computer produces a XEB score between 0 and 1. In the following subsections we characterize the distribution of the XEB score for differently sampled bitstrings.

1. *Distribution of the XEB score of uniformly sampled bitstrings*

A random n-qubit quantum state $|\psi\rangle$ induces a probability distribution on bitstrings $x \in \{0, 1\}^n$. The probabilities $p(x)$ for a random state are assumed to follow the Porter–Thomas distribution with a frequency density $f(p)$:

$$f(p) = N \cdot e^{-N\cdot p}, \tag{III.4}$$

---

[1] If an initial quantum state is present in the server's quantum memory, we can snapshot the classical description of the quantum state which WLOG can be stored in its classical memory since the server (adversary) prepares its own internal quantum memory.

[2] WLOG, this can always be made true. In this case, any processing of the random variables will be deterministic, and any bitstring generated from these random variables alone would be deterministic conditioned on the random variables.

where $N = 2^n$ is the dimension of the Hilbert space.

First, consider a bitstring $x \in \{0,1\}^n$ that is selected uniformly randomly (which we refer to as **uniform sampling**). Denote by $p(x)$ its measurement probability: $p(x) = \mathrm{Tr}(|x\rangle\langle x|\,\rho)$, with $\rho \sim \mathcal{H}(N)$ drawn from ensemble of Haar-random states over n qubits. Even though x is sampled uniformly at random from $\{0,1\}^n$, its corresponding probability, $p(x)$, follows an exponential distribution with the probability density function (PDF) given by

$$\mathrm{PDF}_{x\sim\mathcal{U}}[p(x)] = f(p(x)) = N \cdot e^{-N\cdot p(x)}. \tag{III.5}$$

The sum of m independent random variables drawn from exponential distribution above is given by the Erlang distribution of "shape" m, that is, $\sum_{i\in\mathcal{V}\subset[M]} p_{C_i}(x_i) \sim \mathrm{Erlang}(m,N)$. As a result, for uniform sampling, the cumulative distribution function (CDF) of the XEB score, denoted as $\mathrm{XEB}_{m,\mathcal{U}}$, is given by

$$\Pr\left(\sum_{i\in\mathcal{V}\subset[M]} p_{C_i}(x_i) \le (\chi+1)\cdot m/N\right) = \tilde{\Gamma}(m, m\cdot(\chi+1)) \implies \Pr\left(\mathrm{XEB}_{m,\mathcal{U}} \le \chi\right) = \tilde{\Gamma}(m, m\cdot(\chi+1)), \tag{III.6}$$

where we use $\tilde{\Gamma}$ to denote the regularized lower-incomplete Gamma function [9].

2. *Distribution of the XEB score of bitstrings perfectly sampled from the quantum state*

Second, consider a bitstring x sampled from the distribution corresponding to measurement outcomes of a random quantum state $\rho = |\psi\rangle\langle\psi|$ (which we refer to as **quantum sampling**). The PDF of $p(x)$ is obtained by multiplying the frequency density $f(p(x))$ by the probability of actually measuring the bitstring x, $p(x)$ and by introducing an extra N parameter that renormalizes the distribution:

$$\mathrm{PDF}_{x\sim\mathcal{Q}}[p(x)] = N\cdot f(p(x))\cdot \mathrm{Tr}\left(|x\rangle\langle x|\,\rho\right) = N^2\cdot e^{-N\cdot p(x)}\cdot p(x). \tag{III.7}$$

The sum of m independent random variables with the above PDF given by the Erlang distribution of "shape" $2m$, gives us a CDF on the XEB score for quantum sampling, denoted as $\mathrm{XEB}_{m,\mathcal{Q}}$:

$$\sum_{i\in\mathcal{V}\subset[M]} p(x_i) \sim \mathrm{Erlang}(2\cdot m, N) \implies \Pr\left(\mathrm{XEB}_{m,\mathcal{Q}} \le \chi\right) = \tilde{\Gamma}(2\cdot m, m\cdot(\chi+1)). \tag{III.8}$$

3. *Distribution of the XEB score of bitstrings sampled from a mixture*

If the verification set $\mathcal{V}$ consists of l quantum samples and $m-l$ uniform samples, the sum of probabilities of the quantum samples follows the distribution $\mathrm{Erlang}(2\cdot l, N)$, and the sum of probabilities of the uniform samples has a distribution $\mathrm{Erlang}(m-l, N)$. With a known property of sums of the Erlang distribution [10], the sum of all probabilities therefore has a distribution $\mathrm{Erlang}(m+l, N)$. The CDF of the XEB score, denoted as $\mathrm{XEB}_{m,l}$, is given by

$$\Pr\left(\mathrm{XEB}_{m,l} \le \chi\right) = \tilde{\Gamma}(m+l, m\cdot(\chi+1)). \tag{III.9}$$

4. *Distribution of the XEB score of bitstrings obtained by finite-fidelity quantum sampling*

The effect of a broad class of noise channels on the XEB score is the same as that of depolarizing noise [11, Appendix A]. Therefore, we model any finite-fidelity state as a mixture between the ideal state and the maximally mixed state: $\rho_\phi = \phi\cdot|\psi\rangle\langle\psi| + (1-\phi)\cdot\mathbb{I}/N$, where $\mathbb{I}$ is the identity matrix. Strictly speaking, $1-\phi$ is the depolarizing parameter whose corrections to fidelity, ignored in this work, are exponentially small in the number of qubits. The PDF $p(x)$ from such a finite-fidelity state is given by

$$\begin{aligned}
\mathrm{PDF}_{x\sim\mathcal{Q}_\phi}[p(x)] &= N\cdot f(p(x))\cdot \mathrm{Tr}\left(|x\rangle\langle x|\,\rho_\phi\right)\\
&= N^2\cdot e^{-N\cdot p(x)}\cdot(\phi\cdot p(x) + (1-\phi)/N)\\
&= \phi\cdot N^2\cdot e^{-N\cdot p(x)}\cdot p(x) + (1-\phi)\cdot N\cdot e^{-N\cdot p(x)}\\
&= \phi\cdot \mathrm{PDF}_{x\sim\mathcal{Q}}[p(x)] + (1-\phi)\cdot\mathrm{PDF}_{x\sim\mathcal{U}}[p(x)].
\end{aligned} \tag{III.10}$$

This suggests that the PDF of a fidelity-ϕ sample, which may be generated by sampling a noisy quantum computer or by performing approximate classical simulation, can be interpreted as a stochastic mixture of quantum sampling (Porter–Thomas) and uniform sampling: a bitstring is sampled from the Porter–Thomas distribution with probability ϕ and from the uniform distribution with probability $(1-\phi)$. The probability that ℓ out of m bitstrings are sampled from the Porter–Thomas distribution is given by the binomial distribution. Therefore, the CDF of finite-fidelity XEB, denoted below as $\mathrm{XEB}_{m,\phi}$, is given by

$$\Pr\left(\mathrm{XEB}_{m,\phi} \leq \chi\right) = \sum_{l=0}^{m} \Pr\left(\mathrm{XEB}_{m,\phi} \leq \chi \middle|\, l \text{ PT bitstrings}\right) \cdot \Pr\left(l \text{ PT bitstrings}\right) \tag{III.11}$$

$$= \sum_{l=0}^{m} \tilde{\Gamma}(m+l, m\cdot(\chi+1)) \cdot \left[\binom{m}{\ell}\phi^{l}(1-\phi)^{m-l}\right]. \tag{III.12}$$

C. Adversarial Model and Assumptions

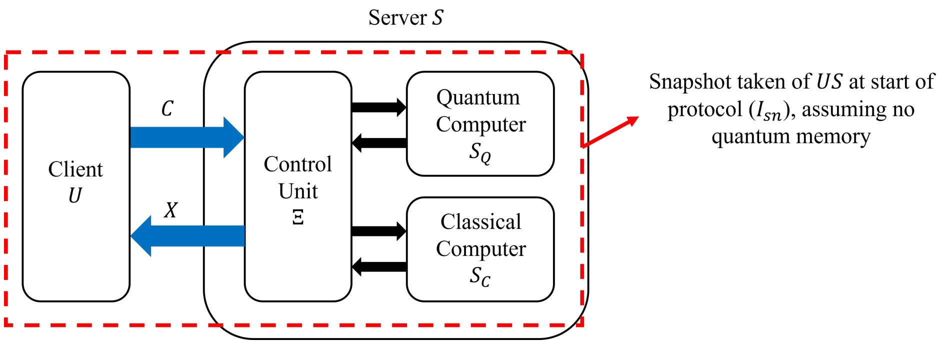

FIG. S2: Model of classical client and malicious server in the certified randomness protocol. The server is split into a classical control unit Ξ, a classical computer S_{C}, and a quantum computer S_{Q}. Without assuming authenticated communication, the server $S = \tilde{S}E$ in general refers to the actual server $\tilde{S}$, along with all parties E that have access to the communication channel between the client and server.

The protocol involves a classical client U, which interacts with a server S with quantum capabilities. The client is assumed to be honest, while the server is treated as the adversary. Let us start by introducing a model of the server S, which consists of a control unit Ξ, classical computer S_{C}, and quantum computer S_{Q}, illustrated in Fig. S2. Without any restriction on the adversarial server, it is clear that any classical server can always pass the XEB test by simulating the circuits. However, it is widely believed that the task of random circuit sampling, commonly used for "quantum supremacy" demonstrations, is hard to perform classically with a reasonable computational power [3, 11–17]. Consequently, by limiting the computing power of the adversary, we can demonstrate that the protocol is sound.

1. *Circuit sampling and hardness assumptions*

The first set of assumptions we make relate to the properties of random circuit sampling and the difficulty in performing random circuit sampling classically, which are widely used in the aforementioned "quantum supremacy" demonstrations.

- The output probabilities of any circuit C belonging to the circuit family follow the Porter–Thomas distribution given in Eq. III.4.
- Achieving high XEB classically is as hard as computing the output probabilities of the quantum circuit for the chosen circuit family;

For the first assumption, extensive numerical evidence exists showing that the output probabilities of random quantum circuits closely follows the Porter–Thomas distribution [3, 8]. For the particular class of circuits considered in this work (see Section IV C), we show that each circuit generates a distribution close to Porter–Thomas in total variation distance (TVD) (see Fig. S3A), with TVD exponentially decaying in n. Additionally, the Shannon entropy of the probability distribution of the random quantum circuits closely matches that of the Porter–Thomas distribution, with the difference vanishing exponentially as well (see Fig. S3B).

The hardness of spoofing XEB is closely related to the hardness of estimating the output distributions of quantum circuits. It has been established that an efficient classical algorithm to estimate output amplitudes of a Haar-random circuit consisting of $m = \mathsf{poly}(n)$ gates to an additive precision $2^{-O(m)}$ would lead to the collapse of the polynomial hierarchy [17, 18]. On the heels of RCS experiments, it was conjectured with the XQUATH (linear cross-entropy threshold assumption) that even estimating the output probabilities of quantum circuits better than the trivial guess of 2^{-n} is difficult, implying the hardness of XEB.

For noisy circuits, Ref. [19] pointed out the limitations of XEB as an evidence of quantum advantage. While prior work implicitly assumed XEB to be a measure of fidelity, it was shown that there are regimes of noise where XEB is no longer a good measure of fidelity and that one can achieve a high XEB despite having a low fidelity (or high noise). Theoretical as well as experimental investigations have since [11, 20] revealed a sharp phase transition between the weak-noise regime where XEB tracks fidelity and the strong-noise regime with a discrepancy between the two, with the low-noise regime characterized as $\epsilon \cdot n < \ln 3 \approx 1.1$ for our architecture [20]. We obtain the value of $\epsilon \cdot n$ for our experiment as follows. We take the simple gate-counting model from [13, Eq. 11], which estimates the fidelity as $\phi_{\mathrm{GC}}(n, d) = \big(1 - \varepsilon(n)\big)^{nd/2}\big(1 - p_{\mathrm{SPAM}}\big)^n$ where $\varepsilon(n)$ is the effective two-qubit gate process infidelity, inclusive of memory errors (which depend on n) and single-qubit gate errors that may be incurred during a layer of circuit execution. Plugging in the estimated 30% overall circuit fidelity in this work obtained from full-circuit mirror-benchmarking experiments, as well as the depth offset of 1.12 and $p_{\mathrm{SPAM}} = 0.00147$ specified in Ref. [13], at $n = 56$ and $d = 10$ we find an effective process infidelity per qubit per layer equal to $\epsilon = \varepsilon(56)/2 \approx 1 - 0.99775$.[3] Since $n = 56$, this gives $\epsilon \cdot n \approx 0.13$, which is well below the critical point of phase transition.

More recently, Ref. [21] proposed a classical algorithm that refutes XQUATH for circuits composed of 2-qubit Haar-random gates and sublinear depth. The same algorithm can also produce bitstrings indistinguishable from a noisy quantum circuit using only polynomially many samples, thus weakening the reliability of XEB for constant noise of any strength. However, the algorithm has a runtime that scales $O(M^{1/\varepsilon})$, where M is the number of samples and ε is the gate infidelity, rendering the algorithm impractical.

Importantly, asymptotic statements do not prove or disprove the difficulty of spoofing finite-sized experiments. The best-known efficient classical algorithms for spoofing XEB either are impractical for realistic experiments or produce an XEB score much lower than that from experiments. Adversaries therefore must resort to inefficient means such as tensor network contraction and approximate circuit simulation. Indeed, the successful spoofs of XEB have come from improved tensor network contraction—with classical computing clusters obtaining an XEB orders of magnitude larger than experiments on a 53-qubit computer. However, we believe that such an attack is unlikely for our experiment on a 56-qubit machine and our measured XEB ≈ 0.32 based on extensive evidence of hardness from [13], which considers the same circuit family.

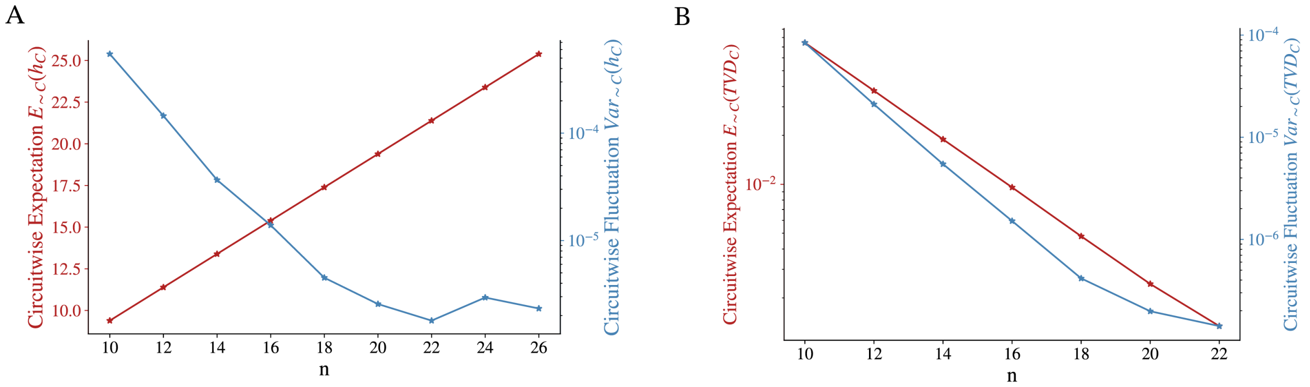

FIG. S3: **Numerical evidence of convergence to Porter–Thomas** (A) Circuit-wise expectation (blue) and variance (red) of Shannon entropy, defined as $-\sum_{x\in\{0,1\}^n} p(x)\log p(x)$ with $p(x) = |\langle x|C|0\rangle|^2$, for distributions induced by $d = 10$ circuits C with different n. (B) Circuit-wise expectation (blue) and variance (red) of Total Variation Distance (TVD) from Porter-Thomas distributions induced by $d = 10$ circuits with different n. To compute the TVD, we convert the vector or probabilities $p(x)$ into a histogram, counting frequencies over discretized probability intervals. Then, we sum the difference in frequency counts so obtained with those expected from Poter-Thomas distribution. For both plots, each data point summarizes 1,000 realizations of random circuits over a fixed two-qubit topology obtained via edge coloring of an n-node graph. Standard error is too small to be visible on the plot.

[3] Substantial improvements made to the H2-1 processor since collection of this experiment's data have since improved the effective process fidelity per qubit per layer to $1 - \varepsilon(56)/2 = 0.9984$ as reported in Ref. [13], not to be confused with the average two-qubit gate fidelity of 0.99843 also reported in that work. However, p_{SPAM} has remained approximately the same.

Consequently we believe that the frugal rejection sampling [22] procedure along with our tensor network contraction simulation method with finite fidelity represents the best-known method that an adversary can utilize, informing the choice of our adversarial model.

2. *Assumptions on computing devices*

Following the model in Fig. S2, we limit the power of the components in the server S, which we recall is composed of a quantum computer S_{Q}, a classical computer S_{C}, and a control unit Ξ. We remind the reader that this set of assumptions is in general imposed on the joint system of the actual server $\tilde{S}$ and any parties that can access the communication channel E, since we do not make the assumption of an authenticated communication channel. The hardness assumption informs our choice of assumption on the power of the classical computer S_{C}:

- The classical computer is capable of $\mathcal{A}$ FLOPS of peak performance.
- The adversary possesses the same methods of tensor network contraction as the client, and that the adversary's classical methods are as equally performant (e.g., precision, numerical efficiency of tensor network contraction) as the client's.
- The classical computer can perform only frugal rejection sampling, which we summarize in Fig. S4. More concretely, it can accept an input circuit C_i and target fidelity $\phi_{\mathcal{A}}^{(i)}$ from Ξ and run the sampling procedure at the target fidelity.

We note that any violation of the second assumption can be captured by increasing the power $\mathcal{A}$ in the first assumption: An adversary possessing more powerful classical methods (higher numerical efficiency given the same contraction scheme, the ability to find contraction schemes with lower costs, the ability to use lower precision, etc.) than the client may be described by absorbing its performance gain into its computational power $\mathcal{A}$.

The output probabilities of the frugal rejection sampling procedure in Fig. S4 are assumed to follow the same distribution as quantum finite-fidelity sampling in Sec. III B 4. We provide evidence for this assumption in Section III C 3. However, recent work [23] argues that one can boost the XEB score of classically obtained samples by postselecting from a large number of low-fidelity samples, leading to a seven-fold improvement for the 2019 Sycamore circuits [3] compared with previous techniques. This effect can be approximately incorporated by absorbing a factor into the adversary computational power $\mathcal{A}$. We did not investigate the improvement factor that could be associated with our experiment. Further, the probability distribution of the XEB score of such an adversary is not understood. Therefore, we leave the analysis of such an adversary for future work.

Input: A single quantum circuit C_i, target classical simulation fidelity $\phi_{\mathcal{A}}^{(i)}$.

Algorithm:

1. Choose a sufficiently large M'.
2. Sample a subset of distinct M' bitstrings $\{x_j\}$ uniformly at random.
3. Calculate all M' probabilities at once by contracting a fraction $\phi_{\mathcal{A}}^{(i)}$ of all slices of the tensor network.
4. For each j: accept x_j with probability $\min\left(1, p(x_j)N/M'\right)$.

Output: The first accepted bitstring x_j.

FIG. S4: The frugal rejection sampling algorithm.

It is difficult to analyze the security of the protocol if the adversary is allowed to perform arbitrary operations on the quantum computer. Thus, we assume a restricted quantum computer S_{Q}.

- The quantum computer is only allowed to execute the circuit, obtaining state $C_i\left|0^n\right\rangle$, and measure it to obtain an n-bitstring X_i with perfect fidelity. More concretely, it can accept the circuit C_i from Ξ, run the circuit C_i, and return the output X_i.
- Every round using the quantum computer (quantum round) is i.i.d.

The second restriction is implied by the first restriction since it specifies exactly how the quantum computer must be used. We also make an assumption of the circuits that are submitted to the quantum computer:

- The choice of circuit for every quantum round is i.i.d.

This assumption is valid when the client selects the circuits in an i.i.d. fashion and Ξ is not choosing which circuits to return quantum samples or which batches to fail in a way that depends on the circuits (no post-selection). We believe assuming no postselection is reasonable for near term adversaries. The argument for this is as follows. For tensor network contraction-based approaches considered for our adversary, the hardness is identical across circuits since the hardness only depends on the circuit topology which stays the same, and only the single-qubit gates change. Additionally, there is no known method for the adversary to say anything about the quality of the sample without running the classical simulation and examine the bitstring probability. If it runs the classical simulation, it might as well use the classical compute budget to provide a classical sample instead of determining post-selection. However, in the future, it will be beneficial to go beyond heuristic arguments and conduct a more rigorous analysis on the possibility of using postselection and understand how much impact that may have.

We note that the quantum computer utilized in the experiment (the honest case) is imperfect and is only able to sample circuits with fidelity $\phi \approx 0.3$. In general, it is possible to assume S_{Q} to be a quantum computer with ability to sample with fidelity *tunable* in $\phi \in [0.3, 1]$. For the purpose of XEB calculations, this corresponds to a probability ϕ of drawing a sample from the PT-distribution and $1 - \phi$ of drawing a sample from the uniform distribution. Since it is straightforward to replace the uniform distribution sample with a known bitstring from its memory device, the entropy contribution from this component in the worst case, conditioned on the snapshot I_{sn}, is 0. In contrast, the samples obtained with simulation in the classical computer S_{C} have a higher $\phi_{\mathcal{A}}^{(i)}$ XEB score contribution also with zero conditional entropy. Since, at a fixed XEB, the conditional entropy is expected to be proportional to $\phi \cdot Q$ (where Q is the number of samples executed on the quantum computer), the server's optimal strategy (for maximizing the average of XEB) is to use the classical computer as much as possible together with the highest-fidelity quantum computer possible (minimum Q). We assume $\phi = 1$ in our analysis for simplicity as a worst-case assumption.

3. *Assumption on frugal rejection sampling*

For the purposes of our calculations, it suffices to show that the probabilities resulting from bitstrings obtained via partial-contraction of tensor networks in which we contract ϕ fraction of slices and the probabilities corresponding to a bitstring obtained by sampling a state depolarized by strength $(1 - \phi)$ are equivalent. To numerically emulate the classical sampling process, we consider a tensor network representation of a n-qubit quantum circuit C, contract $k = \phi \cdot K$ slices, and use the partial amplitudes to sample bitstrings through the frugal rejection sampling process.

Sampling from a depolarized finite fidelity quantum state has the PDF given by Eq. (III.10). However, it is difficult to rigorously derive the PDF associated with samples obtained from partial tensor network contraction. Nevertheless, we numerically observe (see Fig. S5) that such classical samples have probabilities which closely follow Eq. (III.10). This suggests that, for the purposes of our XEB analysis, finite-fidelity classical simulation via partial contraction of tensor networks is equivalent to sampling from a noisy depolarized state.

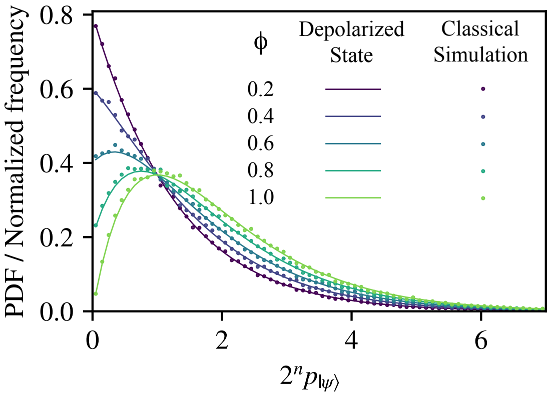

FIG. S5: Distribution of the probability $p_{|\psi\rangle}(x)$. A random circuit with depth 8 acts on $n = 20$ qubits. The tensor network representation is sliced into $K = 1024$ total slices of which we only contract $k = \phi \cdot K$ slices. The partial amplitudes are used to sample bitstrings following the frugal rejection sampling process (Fig. S4). For each ϕ, we sample ten thousand samples via partial contraction (dots).

4. *Adversary strategy restriction*

We restrict the actions of the control unit Ξ and thus the strategy of the adversary. We note that the same restricted adversary is commonly considered in the analysis of certified randomness protocols based on random circuit sampling [11, 24]. In particular, the assumptions are as follows.

- We assume Ξ is not allowed to perform any postselection attacks; in other words, the M detected rounds in the protocol are a fair representation of the adversary's behavior. Therefore, the analysis can be reduced to focus on the M detected rounds;
- We assume that Ξ performs a restricted attack: Among the M detected rounds, Ξ *a priori* selects Q rounds, sends all the circuits C_i for those rounds to the quantum computer, and returns the sample X_i that S_{Q} provides—henceforth called quantum rounds. For the remaining $M-Q$ rounds, it sends C_i and $\phi_{\mathcal{A}}^{(i)}$ to the classical computer and returns the sample X_i that S_{C} provides—-henceforth called classical rounds. We note here that the target fidelity $\phi_{\mathcal{A}}^{(i)}$ may differ between circuits and is dependent on the runtime T_i of each round, as decided by the control unit Ξ;
- For each of the Q rounds, it interacts with the quantum computer only once; that is, it does not attempt to request multiple X_i for the same C_i.

We note here that in general one can consider an arbitrary probabilistic choice of Q. However, since we can describe such an adversary by a convex sum of adversaries with a fixed Q, the satisfaction of soundness for all adversaries with fixed Q would imply that the more general adversary remains sound as well. As also noted in Methods, we point out that these assumptions are likely stronger than necessary and may be remedied in the future.

These assumptions allow us to focus on the M detected rounds, of which Q quantum rounds are a priori selected by the server. A summary of this adversarial model is presented in Fig. S2 and in the main text Fig. 1D. Essentially, this adversary generates the Q samples using a perfect quantum computer, with perfect-fidelity sampling of the Porter–Thomas distribution. For the rest of the $M-Q$ samples, the adversary performs finite-fidelity simulation using frugal rejection sampling. We assume the outputs of frugal rejection sampling are indistinguishable from that obtained by sampling from a finite-fidelity quantum computer as far as XEB statistics are concerned which we justify in Section III C 3, which is equivalent to sampling from the Porter–Thomas distribution with probability $\phi_{\mathcal{A}}^{(i)}$ and the uniform distribution with probability $1-\phi_{\mathcal{A}}^{(i)}$.

The assumptions listed above are necessary for simplification of the security analysis. We leave a general analysis of protocol security to future work. There are some limitations that such a general analysis should address, including (1) the i.i.d. assumption of circuit choice and quantum round, which is inherently at odds with the use of pseudorandom circuit selection, (2) postselection attacks available when we allow batches to be discarded, and (3) oversampling attack. Some of these limitations, such as oversampling, have been previously analyzed in Refs. [11, 24], and one can adapt similar solutions in the general security analysis.

D. Bounds on the Entropy Certified by the Protocol

It is known [25, 26] that a lower bound on the conditional smooth min-entropy of the samples X^M generated through the protocol is sufficient to prove soundness as per Definition 1. In this section we present and prove our main results regarding the amount of smooth min-entropy generated by our randomness certification protocol, deferring the particulars pertaining to soundness and randomness extraction to Section III E.

The min-entropy of a classical-quantum state $\rho_{XA}=\sum_x p(x)\,|x\rangle\!\langle x|\otimes\rho_A^x$ is defined as $H_{\min}(X|A)_\rho=-\log p_{\mathrm{guess}}(X|A)_\rho$, where $p_{\mathrm{guess}}(X|A)_\rho := \sup_{\{M_x\}_x}\sum_x p(x)\,\mathrm{Tr}[\rho_A^x M_x]$, with supremum over POVMs on register A, is known as the guessing probability. The min-entropy can be generalized by including a smoothing parameter $\varepsilon_{\mathrm{s}}\in(0,\sqrt{\mathrm{Tr}[\rho_{XA}]})$ such that

$$H_{\min}^{\varepsilon_{\mathrm{s}}}(X|A)_\rho := \sup_\sigma H_{\min}(X|A)_\sigma\,, \tag{III.13}$$

where the supremum is over states σ_{XA} in the ε_{s}-ball in purified distance centered around ρ, as defined in [27].

The strategy to prove a lower bound on the smooth min-entropy of the samples generated in our protocol proceeds by first bounding the probability that the XEB test passes when the server executes Q quantum rounds, for fixed Q. This bound indirectly allows us to determine $Q_{\min}$, the minimum number of quantum rounds the server has to carry out, from which we can then bound the smooth min-entropy.

Let the client's total computational budget to verify m circuit-sample pairs be $\mathcal{T}$ floating-point operations. The computational cost of simulating a circuit with perfect fidelity is $\mathcal{B}=\mathcal{T}/m$ floating-point operations. In our protocol, the adversary returns

M bitstrings in the non-discarded rounds with a maximum duration of $T_{\text{threshold}} = M \cdot t_{\text{threshold}}$. Let c_{eff} denote the numerical efficiency, which is by assumption equal between the client and the adversary. If the adversary had a large enough classical computer such that $c_{\text{eff}} \cdot \mathcal{A} \cdot T_{\text{threshold}}/(M \cdot \mathcal{B}) \geq 1$, the adversary can simulate all circuits to perfect fidelity and will return classically simulated samples, with confidence that the returned samples will achieve a high XEB score when verified by the client. On the other hand, if $c_{\text{eff}} \cdot \mathcal{A} \cdot T_{\text{threshold}}/(M \cdot \mathcal{B})$ is much less than one, the client cannot simulate all circuits to high fidelity and is thereby compelled to return genuine quantum samples in order to achieve a high XEB score.

The adversary has maximum time $T_{\text{threshold}}$ to return M samples, which is the same time it has to simulate $M - Q$ circuits classically. Assuming a simple but realistic linear model of simulation fidelity [22], we find that, in the worst case, the maximum sum of the simulation fidelities of the $M - Q$ circuits is given by the total executed FLOP count (efficiency times power times maximum time) divided by $\mathcal{B}$:

$$\Phi_{\mathcal{A}} = \sum_{i \text{ classically simulated}} \phi_{\mathcal{A}}^{(i)} = \min\left(M - Q, \frac{c_{\text{eff}} \cdot \mathcal{A} \cdot T_{\text{threshold}}}{\mathcal{B}}\right) = \min\left(M - Q, \frac{c_{\text{eff}} \cdot \mathcal{A} \cdot T_{\text{threshold}}}{\mathcal{T}/m}\right). \tag{III.14}$$

This equation shows that at fixed M and m, the protocol performance should remain the same as long as $\mathcal{A} \cdot T_{\text{threshold}}/\mathcal{T}$ is unchanged. For example, an increase in the adversarial power can be canceled out by a reduction in $T_{\text{threshold}}$ or an increase in $\mathcal{T}$.

As we argue in Section III B, finite-fidelity classical bitstrings can be interpreted as being drawn from the Porter–Thomas distribution with probability $\phi_{\mathcal{A}}^{(i)}$ and from the uniform distribution with probability $1 - \phi_{\mathcal{A}}^{(i)}$. The total number of Porter–Thomas bitstrings is therefore a random number. Let Z_i be the indicator random variable such that among samples classically simulated, the sample i is drawn from the Porter–Thomas distribution if and only if $Z_i = 1$ and from $Z_i = 0$ otherwise. The random variable L_{C} representing the total number of Porter–Thomas bitstrings in $M - Q$ classical samples is therefore

$$L_{\text{C}} = \sum_{i \text{ classically simulated}} Z_i, \tag{III.15}$$

and its expected value is, in the worst case, given by

$$\mathbb{E}[L_{\text{C}}] = \sum_{i \text{ classically simulated}} \phi_{\mathcal{A}}^{(i)} = \Phi_{\mathcal{A}}. \tag{III.16}$$

Recalling that we assume the server is i.i.d., we use Chernoff's inequality for sum of Bernoulli random variables [28] to obtain an upper bound on the probability that L_C exceeds $L_{\text{C,max}} := (1 + \delta) \cdot \mathbb{E}[L_{\text{C}}]$ for some $\delta > 0$:

$$\Pr\left[L_{\text{C}} \geq L_{\text{C,max}}\right] \leq \exp\left(-\frac{\delta^2 \cdot \mathbb{E}[L_{\text{C}}]}{3}\right) = \varepsilon_1 \implies L_{\text{C,max}} = \Phi_{\mathcal{A}}\left(1 + \sqrt{\frac{3}{\Phi_{\mathcal{A}}} \ln \frac{1}{\varepsilon_1}}\right). \tag{III.17}$$

Since all quantum samples have fidelity one and are Porter–Thomas bitstrings, we can obtain an upper bound L_{max} on the random variable representing the total number of Porter–Thomas bitstrings L in <u>all</u> M samples (both quantum and classical):

$$L = Q + L_{\text{C}} \implies \Pr\left[L \geq L_{\text{max}} = Q + L_{\text{C,max}}\right] \leq \varepsilon_1. \tag{III.18}$$

Since the client performs verification only on a much smaller set of circuits $\mathcal{V}$ with $|\mathcal{V}| = m$, the distribution of the XEB score over the verification set depends on the number of Porter–Thomas samples in the verification set, not the number of Porter–Thomas samples in total. Each of the m verification samples is drawn from a total of M samples without replacement, and up to L_{max} of the M samples follow the Porter–Thomas distribution. If L_{max} out of M samples are Porter–Thomas, then the probability that l out of m samples are Porter–Thomas is given precisely by the hypergeometric distribution Hypergeometric(M, L_{max}, m) [29].

We can now obtain the probability of the adversary achieving measured XEB score below threshold χ as

$$\Pr\left[\text{XEB}_{\text{test}} \leq \chi | L = L_{\text{max}}\right] = \sum_{l=0}^{m} \Pr\left[\text{XEB}_{\text{test}} \leq \chi | l \text{ PT bitstrings in } \mathcal{V}\right] \cdot \Pr\left[l \text{ PT bitstrings in } \mathcal{V} | L = L_{\text{max}}\right] \tag{III.19}$$

$$= \sum_{l=0}^{m} \tilde{\Gamma}(m + l, m \cdot (\chi + 1)) \cdot \left[\binom{M}{m}^{-1} \binom{m}{l} \binom{M - L_{\text{max}}}{m - l}\right] = 1 - \varepsilon_2, \tag{III.20}$$

where $\tilde{\Gamma}$ is the regularized lower-incomplete Gamma function that, as we discuss in Section III B, gives the distribution of XEB with a mixture of uniform and Porter–Thomas bitstrings. Here, we remark that Eq. (III.20) holds when the choice of testing subset $\mathcal{V} \subset [M]$ of size m is random. When the choice of $\mathcal{V}$ is pseudorandom, then $\Pr\left[\text{XEB}_{\text{test}} \leq \chi | L = L_{\text{max}}\right]$ is negligibly

close to Eq. (III.20). Putting it all together, the probability that an adversary sampling the quantum computer Q times attains a threshold χ is

$$\begin{aligned}\Pr[\Omega] &= \Pr\left[\mathrm{XEB}_{\mathrm{test}} \geq \chi\right] & \text{(III.21)}\\ &= \Pr\left[L > L_{\max}\right]\Pr\left[\mathrm{XEB}_{\mathrm{test}} \geq \chi \,|\, L > L_{\max}\right] & \text{(III.22)}\\ &\quad + \Pr\left[L \leq L_{\max}\right]\Pr\left[\mathrm{XEB}_{\mathrm{test}} \geq \chi \,|\, L \leq L_{\max}\right] & \text{(III.23)}\\ &\leq \Pr\left[L > L_{\max}\right] + \Pr\left[\mathrm{XEB}_{\mathrm{test}} \geq \chi \,|\, L \leq L_{\max}\right] & \text{(III.24)}\\ &\leq \varepsilon_1 + \varepsilon_2, & \text{(III.25)}\end{aligned}$$

with ε_1 and ε_2 as in Eq. (III.18) and Eq. (III.20), respectively. Here, the last inequality follows from the fact that for all $l \leq L_{\max}$ $\Pr\left[\mathrm{XEB}_{\mathrm{test}} \geq \chi \,|\, L = l\right] \leq \Pr\left[\mathrm{XEB}_{\mathrm{test}} \geq \chi \,|\, L = L_{\max}\right]$, which consequently implies

$$\begin{aligned}\Pr\left[L \leq L_{\max}\right]\Pr\left[\mathrm{XEB}_{\mathrm{test}} \geq \chi \,|\, L \leq L_{\max}\right] &= \sum_{l \leq L_{\max}} \Pr\left[L = l\right]\Pr\left[\mathrm{XEB}_{\mathrm{test}} \geq \chi \,|\, L = l\right] & \text{(III.26)}\\ &\leq \sum_{l \leq L_{\max}} \Pr\left[L = l\right]\Pr\left[\mathrm{XEB}_{\mathrm{test}} \geq \chi \,|\, L = L_{\max}\right] & \text{(III.27)}\\ &= \Pr\left[\mathrm{XEB}_{\mathrm{test}} \geq \chi \,|\, L = L_{\max}\right]. & \text{(III.28)}\end{aligned}$$

We summarize this result in the following lemma.

Lemma 2. *Let Ω be the event that an adversary, with a classical computational power of $\mathcal{A}$ and samples Q out of M samples on a perfect-fidelity quantum computer, passes the XEB test with threshold χ. We have $\Pr[\Omega] \leq \varepsilon_{\mathrm{adv}}(Q,\chi) = \varepsilon_1 + \varepsilon_2$, for*

$$\varepsilon_1 = \exp\left(-\frac{\delta^2 \cdot \Phi_{\mathcal{A}}}{3}\right) \qquad \varepsilon_2 = 1 - \sum_{l=0}^{m} \tilde{\Gamma}(m+l, m\cdot(\chi+1)) \cdot \left[\binom{M}{m}^{-1}\binom{m}{l}\binom{M-(Q+\Phi_{\mathcal{A}}(1+\delta))}{m-l}\right], \qquad \text{(III.29)}$$

$\Phi_{\mathcal{A}}$ given by Eq. (III.14), and $\delta \geq 0$.

Given this lemma, an XEB threshold χ, and a target $\varepsilon_{\mathrm{s}} \in (0, 1/4)$, we can compute $Q_{\min} = \arg\min_Q\{\varepsilon_{\mathrm{adv}}(Q,\chi) \geq 4\varepsilon_{\mathrm{s}}\}$, which allows us to bound the smooth min-entropy of the samples X_M conditioned on side-information $\tilde{I}_{\mathrm{sn}} = K_{\mathrm{seed}}S^0$, the initial snapshot I_{sn} excluding the randomness extractor seed K_{ext}. The prescription of how to solve for $Q_{\min}$ using this lemma is provided in Section IV F. Formally we have the following theorem:

Theorem 3. *Let Ω denote the event where the randomness certification protocol in Figure 4 of Methods does not abort, and let σ be the state over registers X^M and $\tilde{I}_{\mathrm{sn}}$ prior to the randomness extraction phase of the protocol. Given $\varepsilon_{\mathrm{s}} \in (0, 1/4)$, the protocol either aborts with probability greater than $1 - 4\varepsilon_{\mathrm{s}}$ or*

$$H_{\min}^{\varepsilon_{\mathrm{s}}}(X^M|\tilde{I}_{sn})_{\sigma\wedge\Omega} \geq Q_{\min}(n-1) - \log\frac{1}{\varepsilon_{\mathrm{s}}}, \qquad \text{(III.30)}$$

where $\sigma = \sigma_{X^M|\tilde{I}_{sn}}$ is the state prior to the randomness extraction stage and $Q_{\min} = \arg\min_Q\{\varepsilon_{\mathrm{adv}}(Q,\chi) \geq 4\varepsilon_{\mathrm{s}}\}$ for $\varepsilon_{\mathrm{adv}}(Q,\chi)$ as in Lemma 2.

Proof. Denoting the initial snapshot as $\tilde{I}_{\mathrm{sn}}$, consider the smooth-min-entropy $H_{\min}^{\varepsilon_{\mathrm{s}}}(X^M|\tilde{I}_{\mathrm{sn}})_{\sigma\wedge\Omega}$ conditioned on the snapshot. Note that the choice of smoothing parameter ε_{s} is valid since $\mathrm{Tr}\left[\sigma_{X^M\tilde{I}_{\mathrm{sn}}\wedge\Omega}\right] \geq 4\varepsilon_{\mathrm{s}}$ and $0 < \varepsilon_{\mathrm{s}} < 2\sqrt{\varepsilon_{\mathrm{s}}}$ which implies $H_{\min}^{\varepsilon_{\mathrm{s}}}(X^M|\tilde{I}_{\mathrm{sn}})_{\sigma\wedge\Omega} \leq H_{\min}^{2\sqrt{\varepsilon_{\mathrm{s}}}}(X^M|\tilde{I}_{\mathrm{sn}})_{\sigma\wedge\Omega}$. The adversary's strategy is to return quantum samples for Q out of M total samples. For any choice of Q, we have

$$H_{\min}^{\varepsilon_{\mathrm{s}}}(X^M|\tilde{I}_{\mathrm{sn}})_{\sigma\wedge\Omega} \geq H_{\min}^{\varepsilon_{\mathrm{s}}}(X_Q|\tilde{I}_{\mathrm{sn}})_{\sigma\wedge\Omega} \geq H_{\min}^{\varepsilon_{\mathrm{s}}}(X_Q|\tilde{I}_{\mathrm{sn}})_{\sigma}, \qquad \text{(III.31)}$$

where the first inequality follows since the registers X^M are classical and the second from [26, Proposition 10]. Now, since all of the registers in the last quantity are classical and produced in an i.i.d. manner (once the global event Ω is removed), we can use [30, Equation 5] to obtain

$$H_{\min}^{\varepsilon_{\mathrm{s}}}(X_Q|\tilde{I}_{\mathrm{sn}})_{\sigma} \geq Q \cdot H_2(X_i|C_i)_{\sigma} - \log\frac{1}{\varepsilon_{\mathrm{s}}} = Q\cdot(n-1) - \log\frac{1}{\varepsilon_{\mathrm{s}}}, \qquad \text{(III.32)}$$

where i is an arbitrary choice of index in $[Q]$ (since the Q rounds are i.i.d.) and we have noted that the only available side information in quantum rounds is the choice of circuit C_i (computable from $\tilde{I}_{\text{sn}}$). Further, $H_2(X) := -\log \sum_x p(x)^2$ is the 2-Renyi entropy (or collision entropy), which for the Porter–Thomas distribution is

$$H_2(X_i|C_i)_\sigma = -\log_2 \left[\int_0^\infty N \cdot f(p) \cdot p^2 \cdot \mathrm{d}p\right] = n - 1. \tag{III.33}$$

Given $\Pr[\Omega]_\sigma \geq 4\varepsilon_{\text{s}}$, the minimum Q that any adversary strategy can have is precisely

$$Q_{\min} = \min\{Q \,:\, \varepsilon_{\text{adv}}(Q, \chi) \geq 4\varepsilon_{\text{s}}\}, \tag{III.34}$$

with $\varepsilon_{\text{adv}}(Q, \chi)$ defined in Lemma 2. This immediately gives

$$H_{\min}^{\varepsilon_{\text{s}}}(X^M|\tilde{I}_{\text{sn}})_\sigma \geq Q_{\min}(n-1) - \log\frac{1}{\varepsilon_{\text{s}}}, \tag{III.35}$$

as required. □

E. Proof of Protocol Soundness

Recalling Definition 1, to prove ε_{sou}-soundness for some $\varepsilon_{\text{sou}} \in (0, 1]$, we want to show that

$$\|\rho_{KI_{\text{sn}}\wedge\Omega} - \tau_K \otimes \rho_{I_{\text{sn}}\wedge\Omega}\|_{\text{Tr}} \leq \varepsilon_{\text{sou}}\,, \tag{III.36}$$

where Ω is the event that the protocol does not abort, K is the register containing the ℓ-bitstring output by the seeded extractor, and I_{sn} is the snapshot, composed of the client's (uniform and secret) extractor seed K_{ext}, random bitstring K_{seed}, and the snapshot of the server's memory S^0 (which, by definition, includes both server and the environment). Further, $\rho_{KI_{\text{sn}}\wedge\Omega}$ is the state at termination of the randomness certification protocol and $\tau_K \otimes \rho_{I_{\text{sn}}\wedge\Omega}$ is the ideal functionality of the protocol, which replaces ρ_K with the maximally mixed state $\tau_K = \frac{1}{2^\ell}\sum_{i\in[2^\ell]} |i\rangle\langle i|$.

We begin by defining the class of randomness extractors used in our randomness certification analysis: quantum-proof strong extractors [25, 26, 31–33].

Definition 4 (Quantum-proof strong extractor [25, 31, 32]). *A function Ext : $\{0,1\}^n \times \{0,1\}^s \to \{0,1\}^\ell$ is a quantum-proof strong $(\kappa, \varepsilon_{\text{ext}})$-extractor if, for any classical quantum state ρ_{SE} where S is the classical register with dimension 2^n for which $H_{\min}^{\varepsilon_{\text{s}}}(S|E)_\rho \geq \kappa$, it holds that*

$$\|\mathit{Ext}(\rho_{SE} \otimes \tau_D) - \tau_K \otimes \tau_D \otimes \rho_E\|_{\text{Tr}} \leq \frac{1}{2}\varepsilon_{\text{ext}} + 2\varepsilon_{\text{s}}, \tag{III.37}$$

where τ_D, and τ_K are maximally mixed state with dimension 2^s and 2^ℓ respectively and $\varepsilon_{\text{s}} \in \left[0, \sqrt{\text{Tr}[\rho_{SE}]}\right]$. The map Ext acts on the classical systems S and D. The input on system D is called the seed of the extractor.

In our protocol, the S-register is precisely the M classical registers $X_1, \ldots, X_M$, and the D register is K_{ext} and contains the s-bit random seed of the extractor that is private to the client. The output length of the extractor depends on the amount of entropy of the input as well as ε_{ext} and the seed length. For example, if we use a 2-universal strong extractor, then we get the following bound on ℓ.

Lemma 5 (2-Universal Strong Extractor [25]). *There exist a quantum-proof strong $(\kappa, \varepsilon_{\text{ext}})$-extractor Ext : $\{0,1\}^{nM} \times \{0,1\}^s \to \{0,1\}^\ell$ with seed length $s = nM$ and $\ell \leq \kappa - 2\log\left(\frac{1}{\varepsilon_{\text{ext}}}\right)$.*

Alternatively we can use strong extractors requiring a shorter seed at the cost of a smaller output.

Lemma 6 (Trevisan Extractor [33]). *There exist a quantum-proof strong $(\kappa, \varepsilon_{\text{ext}})$-extractor Ext : $\{0,1\}^{nM} \times \{0,1\}^s \to \{0,1\}^\ell$ with seed length $s = O(\log(nM))$ and $\ell \leq \kappa - 4\log\left(\frac{1}{\varepsilon_{\text{ext}}}\right) - 4\log\ell - 6$.*

In the following, we show that the smooth min-entropy bound provided in Theorem 3 can be used to guarantee ε_{sou}-soundness when using a strong randomness extractor.

Corollary 7. *Let $\varepsilon_{\rm sou} \in (0,1]$, and suppose that a $(\kappa, \varepsilon_{\rm sou})$-quantum-proof strong extractor is used in the randomness extraction step in the randomness certification protocol, where*

$$\kappa = Q_{\min}(n-1) - \log \frac{1}{\varepsilon_{\rm sou}} - 2 \tag{III.38}$$

and where $Q_{\min} = \min\{Q : \varepsilon_{\rm adv}(Q, \chi) \geq \varepsilon_{\rm sou}\}$. Then, the protocol is $\varepsilon_{\rm sou}$-sound.

In particular, the protocol is $\varepsilon_{\rm sou}$-sound if a two-universal hash function (e.g., Toeplitz hashing) is utilized and the length of the output satisfies

$$\ell \leq Q_{\min}(n-1) - 3\log \frac{1}{\varepsilon_{\rm sou}} - 2. \tag{III.39}$$

Alternatively, the protocol is $\varepsilon_{\rm sou}$-sound if a Trevisan extractor is utilized and the length of the output ℓ satisfies

$$\ell \leq Q_{\min}(n-1) - 5\log \frac{1}{\varepsilon_{\rm sou}} - 4\log \ell - 8. \tag{III.40}$$

Proof. We begin by considering the trace distance $\left\|\rho_{KK_{\rm ext}\tilde{I}_{\rm sn}\wedge\Omega} - \tau_K \otimes \tau_{K_{\rm ext}} \otimes \rho_{\tilde{I}_{\rm sn}\wedge\Omega}\right\|_{\rm Tr}$, where $\tilde{I}_{\rm sn}$ is the initial snapshot $I_{\rm sn}$ excluding the randomness extractor seed $K_{\rm ext}$. In the case where $\Pr[\Omega]_\rho < \varepsilon_{\rm sou} = 4\varepsilon_{\rm s}$,

$$\left\|\rho_{KK_{\rm ext}\tilde{I}_{\rm sn}\wedge\Omega} - \tau_K \otimes \tau_{K_{\rm ext}} \otimes \rho_{\tilde{I}_{\rm sn}\wedge\Omega}\right\|_{\rm Tr} \leq \frac{1}{2}\left\|\rho_{KK_{\rm ext}\tilde{I}_{\rm sn}\wedge\Omega}\right\|_1 + \frac{1}{2}\left\|\tau_K \otimes \tau_{K_{\rm ext}} \otimes \rho_{\tilde{I}_{\rm sn}\wedge\Omega}\right\|_1 = \Pr[\Omega]_\rho < \varepsilon_{\rm sou}, \tag{III.41}$$

and soundness is automatically guaranteed. In the case where $\Pr[\Omega]_\rho \geq \varepsilon_{\rm sou} = 4\varepsilon_{\rm s}$, Theorem 3 implies

$$H_{\min}^{\varepsilon_{\rm s}}(X^M|\tilde{I}_{\rm sn})_{\sigma_{\wedge\Omega}} \geq Q_{\min}(n-1) - \log\frac{1}{\varepsilon_{\rm s}} = Q_{\min}(n-1) - \log\frac{1}{\varepsilon_{\rm sou}} - 2. \tag{III.42}$$

Setting $\kappa = Q_{\min}(n-1) - \log\frac{1}{\varepsilon_{\rm sou}} - 2$, Definition 4 of $(\kappa, \varepsilon_{\rm sou})$-quantum-proof strong extractors implies

$$\left\|\rho_{KK_{\rm ext}\tilde{I}_{\rm sn}\wedge\Omega} - \tau_K \otimes \tau_{K_{\rm ext}} \otimes \rho_{\tilde{I}_{\rm sn}\wedge\Omega}\right\|_{\rm Tr} \leq \frac{1}{2}\varepsilon_{\rm sou} + 2\varepsilon_{\rm s} = \varepsilon_{\rm sou}, \tag{III.43}$$

provided the length ℓ of the output string K satisfies either

$$\ell \leq \kappa - 2\log\frac{1}{\varepsilon_{\rm sou}}, \tag{III.44}$$

as per Lemma 5 if a 2-universal extractor is used, or

$$\ell \leq \kappa - 4\log\frac{1}{\varepsilon_{\rm sou}} - 4\log\ell - 6, \tag{III.45}$$

as per Lemma 6 if a Trevisan extractor is used. This concludes the proof. □

F. Randomness Expansion

As introduced in Methods, the protocol generally requires some initial randomness, and we ideally like the output of the protocol (the extracted bits together with the seed used by the extractor) to be larger than the required randomness input. Here, we examine in detail the amount of randomness expansion of a single protocol run, although the actual expansion rate also depends on assumptions on the source of initial randomness and the output string K. In the worst case, the expected increase in randomness can be given by

$$\ell_{\rm expand} = \Pr[\Omega](\ell - |K_{\rm ext}|) - r \tag{III.46}$$

with the terms being the following:

1. The expected length of random bitstring generated, $\Pr[\Omega]\ell$, noting that the protocol returns $K = \perp$ when the protocol aborts;

2. The expected length of the randomness extractor seed utilized, $\Pr[\Omega]|K_{\text{ext}}|$, noting that the randomness extraction is not performed when the protocol aborts;

3. The length of seed required to generate the circuits and to select the test rounds, r. We note that this randomness is utilized even when the protocol aborts.

The assumptions on the sources of the random string can impact the terms to consider in ℓ_{expand}. For instance, the source of random strings can be from sources that are not certified, but trusted to be random and inaccessible to the adversary (e.g., public randomness beacon announcing random bits after the server's response). The consideration here would be that the client sacrifices randomness that the client is unable to certify in order to gain randomness that is certified. Whether this assumption is suitable depends on the context of the protocol usage.

Separately, we note that the extractor seed cannot be reused in general, unlike in some QKD protocols where the seed is preshared (not announced). The reason is that the input to the extractor, X^M, is public and known to the server. Since K is a deterministic function of X^M and K_{ext}, the usage of K could result in reveal of the seed K_{ext}. If we assume that K is never utilized in any public manner, in other words, is used only internally by the client for applications that would not leak K externally, K_{ext} remains private and can be reused. This is the assumption we use in the main text of the paper to claim randomness expansion.

IV. Details of Protocol Implementation

We now provide additional details on the engineering challenges and implementation choices associated with our experiment.

A. Quantum Circuit Simulation by Tensor Network Contraction

A tensor is a collection of indexed numbers, which can graphically be represented by a node with an edge or "bond" leaving the node for every index. A tensor network is a graph representation of an equation corresponding to the product of many tensors, where edges/bonds shared between nodes in the graph correspond to dummy indices that are summed over in the product. Open indices that remain unsummed can belong only to one tensor and typically represent physical degrees of freedom in the context of quantum simulation. The process of calculating the output of a tensor network by iteratively summing over each dummy index is called "contraction" of the tensor network.

Tensor networks have a wide range of applications including quantum simulation, quantum computation, quantum control, and machine learning [34, 35]. In the context of quantum simulation, there are two leading algorithms for simulating random circuits, and both are based on tensor networks. The first approach constructs a tensor network corresponding to the product of unitary matrices in the quantum circuit and contracts the network. The second approach constructs an approximate quantum state using a tensor network and approximately time-evolves it according to the quantum circuit. As shown in Ref. [13], achieving fidelities and runtimes comparable to the quantum computer used in this demonstration on circuits of the structure we consider appears to be well beyond state-or-the-art implementations of known algorithms for exact tensor network contraction. Moreover, one can frustrate approximate tensor-network methods considerably more deeply by slight modifications to the single-qubit gate set of our circuits, without impacting the verification time. A similar conclusion was reached in Ref. [11] for random circuit sampling on a two-dimensional grid of qubits. Therefore, in this work we focus on analyzing the first approach and refer the reader to Ref. [13] for a detailed comparison of simulation techniques.

A quantum circuit has a corresponding unitary matrix that can be expressed as matrix multiplications of individual quantum gates. One can write the unitary matrix of the quantum circuit as a contraction equation of individual gate unitaries and graphically represent it as a tensor network. If we fix the values of the indices of all input qubits to be zero and output qubits to the bits in bitstring x, respectively, then contracting the tensor network gives the transition amplitude $\langle x|U|0\rangle$. The order in which indices are contracted can change the computational cost of performing the contraction dramatically, since different contraction orders lead to different intermediate tensor sizes.

1. *Index slicing*

Even with an optimized contraction path, the dimension of the largest intermediate tensor may be so large that it cannot be fit into a single graphics processing unit (GPU). As a result, Ref. [36] introduced index slicing, where tensor network indices are removed from the computation by fixing them to certain values. A sliced tensor network can then be contracted with lower memory overhead. This process is repeated for all possible values of the removed indices. For example, if two indices are sliced, we construct four tensor networks that correspond to those indices fixed to $\{0,0\}$, $\{0,1\}$, $\{1,0\}$, $\{1,1\}$. Each tensor network is

GPU type	TFLOPS per GPU (FP32)	Time to simulate one circuit (10^6 seconds)	Flop count per circuit (10^{18})	Efficiency
AMD MI250X	53	3.5	90	49%
NVIDIA V100	14	3.9	35	64%
NVIDIA A100	19.5	3.0	35	60%

TABLE I: GPU performance of AMD MI250X (Frontier [40]), NVIDIA V100 (Summit [41]), and NVIDIA A100 (Perlmutter [42] and Polaris [43]). Each AMD MI250X GPU has 2 Graphics Compute Dies (GCDs). Each GCD has a theoretical peak performance of 26.5 teraFLOPS (TFLOPS) for double and single precision.

called a slice and is contracted before the scalar values are aggregated to give the final result of contraction of the original tensor network. If only a fraction $\Phi_{\mathcal{A}}$ of slices are contracted, the simulation fidelity is approximately $\Phi_{\mathcal{A}}$ [22].

2. *Trade-off between compute and memory operations*

The joint optimization of the contraction order and the choice of which indices to slice have a significant impact on the total floating-point operations required, the numerical efficiency, and the memory requirement of performing a tensor network contraction [11, 37, 38]. Moreover, optimizing the tensor network contraction order generally presents a trade-off between these metrics. Optimization generally aims to minimize the number of FLOPs required, but contraction schemes with low FLOP count may be highly inefficient. For example, these schemes might involve contractions between a large tensor and a very small tensor. Such a contraction has low ratio of compute (FLOP count) to data movement (limited by memory bandwidth), which is referred to as arithmetic intensity. As a result, the optimization is usually performed with respect to some combined objective function that balances FLOP count with data movement.

3. *Simulation algorithm in our experiment*

We now present the techniques used to estimate the circuit simulation cost in Sec. IV C and to perform verification in Sec. IV E. We use the tensor-network optimizer package "CoTenGra" [39] to determine the contraction scheme. We refer the reader to Ref. [13] for a detailed comparison of various methods, which shows that CoTenGra is one of the best tensor-network optimization packages among those that are suitable for single-amplitude contractions. We note that since we are obtaining one amplitude or one sample per circuit, intermediate tensor caching and reuse does not improve performance.

Within CoTenGra, we perform contraction order optimization using "KaHyPar" as the primary method. We then perform slicing by interleaving slicing and subtree reconfiguration as well as simulated annealing. This process ensures that the largest intermediate tensor has 28 indices, to limit the amount of overall memory demanded to store intermediate states of the contractions.

Each contraction scheme has a certain number of slices with a certain FLOP count. Given a GPU with a known computational power (FLOPs per second, or FLOPS), the time it takes for a GPU to contract a slice is at least the FLOP count per slice divided by the theoretical peak performance of the GPU. We denote this ratio the theoretical contraction time. In practice, the actual time it takes to perform contraction on a GPU differs from the estimate we obtain from the optimized contraction order; the ratio of theoretical contraction time and the actual contraction time is referred to as *efficiency*. We observe that the efficiency of contraction also depends on how much we account for memory operations in the optimization of contraction order. In CoTenGra, the memory-weighted cost is referred to as "combo-α," where α is the weighing factor for memory operations. In the limit where we do not take memory operations into account ($\alpha \to 0$), the contraction order is optimized to minimize only the FLOP count. We observe that higher α increases the efficiency at the expense of a potentially higher FLOP count.

To find the best contraction scheme that results in the shortest actual time on GPUs, we optimize the contraction order at different α and measure the actual contraction time and efficiency on different GPUs. As an example, the α-dependence on V100 GPUs is shown in Fig. S6. The best contraction orders for different GPUs are different. Overall, we produce a total of $\approx$ 750k contraction schemes at different α values. For each GPU, we pick the contraction scheme with the lowest time-to-solution for actual verification. Different GPUs have different optimal contraction schemes with different FLOP counts and efficiencies, which we report in Table I. In practice, however, the FLOP counts can be higher than the theoretical estimate due to some additional tensor manipulations. For NVIDIA A100, the total FLOPs for a single circuit is 36.6×10^{18} when benchmarked using NVIDIA Nsight Compute, which corresponds to an efficiency of 63%, slightly higher than the 60% efficiency estimated using CoTenGra FLOP count.

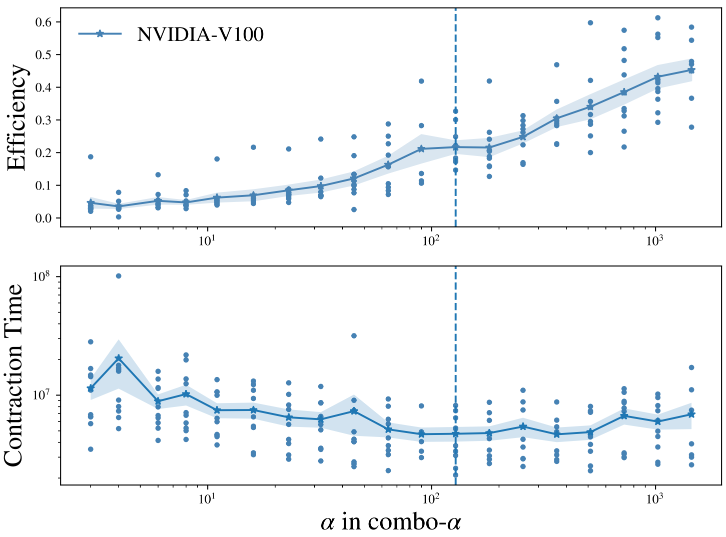

FIG. S6: Contraction performance, in terms of efficiency (top) and time (bottom), of ten depth-10 circuits on NVIDIA-V100, as a function of α, the weight parameter in the objective of contraction order. The blue shaded region around blue line (mean) represents standard error. The vertical dashed line represents $\alpha = 128$, chosen for contraction on Summit.

4. *Heterogeneous high-performance computing platforms of our experiment*

We use four U.S. Department of Energy supercomputers to perform quantum circuit simulation for verification: Frontier and Summit at the Oak Ridge Leadership Computing Facility, Perlmutter at the National Energy Research Scientific Computing Center, and Polaris at the Argonne Leadership Computing Facility. The technical specifications needed to calculate the single-precision theoretical peak performance (number of FLOPS) of the supercomputers are listed in Table II, and we estimate the time to simulate one circuit as well.

The actual time-to-solution on any given supercomputer depends on numerous factors in addition to the theoretical peak performance. Some computational tasks may be more compute-heavy and are bottlenecked by the theoretical peak performance, whereas others may be more memory operations-heavy and are bottlenecked by the memory bandwidth. The device architecture also affects the ability for the tensor network contraction to utilize caching, reduced or mixed precision, and other techniques. The software stack may also affect the ability to use just-in-time compilation, efficiency of high-dimensional tensor manipulation, specialized math libraries, and proprietary quantum simulation software such as cuQuantum by NVIDIA. For example, while we use CoTenGra for contraction scheme optimization, we use cuQuantum to perform actual contractions on NVIDIA GPU supercomputers and CoTenGra with the JAX backend on Frontier. As a result, the only reliable way to determine the cost of a computational task is to profile the actual time-to-solution on the device instead of relying on metrics such as FLOP count or total data movement cost.

Consider the computational cost of the actual quantum circuits executed on the quantum device to generate certified randomness. In Sec. IV C we explain how we chose the circuits. For a unified and simple understanding of the computational budget and computational power, we convert the overall supercomputing budget on various supercomputers into Frontier node-hours. The lowest time-to-solution contraction scheme on Frontier, after balancing FLOP count and memory operations, would take 3.5 million seconds to be simulated on a single MI250X GPU. Therefore, simulating a single circuit takes one Frontier node $3.5 \times 10^6/(4 \times 3600) \approx 243$ hours. One Frontier node-hour thus corresponds to $1/243$ of the cost of a single circuit.

To convert our budget on other supercomputers into Frontier node-hours, we find the lowest time-to-solution contraction schemes on these supercomputers. We multiply 243 by the number of simulated circuits (1522) to get the effective Frontier node-hours we have for validation. We estimate a total of approximately 370k effective Frontier node-hours across all of the supercomputers. This corresponds to $370000 \times 3600 \times 53 \times 10^{12} = 2.8 \times 10^{23}$ theoretical floating-point operations on Frontier. The contraction scheme on Frontier corresponds to a FLOP count of $\mathcal{B} \approx 10^{20}$ per circuit and can be executed with an efficiency of $c_{\text{eff}} \approx 50\%$. We remark that a loose upper bound on maximum achievable Frontier efficiency on a realistic application is provided by the LINPACK performance benchmark used by the TOP500 list [44], on which Frontier achieved only 71.1% efficiency. We further note that efficiency at full-machine scale that we measure and report in Table III is slightly lower than single-node efficiency. However, since the efficiency decay is small and comparable across all supercomputers utilized in this work, we use single-node benchmarking data for the purpose of node-hour conversion and total budget estimation.

We emphasize that we do not perform exactly 2.8×10^{23} floating point operations. One obvious reason is that the efficiency of

	Compute nodes	GPU type	GPUs per node	PFLOPS (FP32)
Frontier	9408	AMD MI250X	4	1994
Summit	4608	NVIDIA V100	6	387
Perlmutter	1536	NVIDIA A100	4	120
Polaris	560	NVIDIA A100	4	44

TABLE II: Supercomputer technical specifications for Frontier [40], Summit [41], Perlmutter [42], and Polaris [43]. All FLOPS listed are for single precision (FP32). Total performance in petaFLOPS (PFLOPS) is the theoretical peak performance obtained by multiplying the number of GPUs and the single GPU performance, available in Table I.

Frontier is not 100%. A different reason is that any given contraction scheme on a different supercomputer will have a different FLOP count and efficiency. Even the same contraction scheme has different efficiencies on different supercomputers. This is why when computing effective Frontier node-hours, we use only the time-to-solution, and we measure our budget using effective Frontier FLOP count and use the efficiency on Frontier. Similarly, we report the cost of simulating quantum circuits using the FLOP count and efficiency on Frontier.

B. Selection of Experimental Parameters

The success of our protocol, outlined in the main text and in the overview in Sec. II, depends on the careful choice of challenge circuits: classical simulation of these circuits must be difficult enough to preclude fast classical simulation by the adversary and easy enough such that client-side verification is possible with a supercomputer. Since in practice we have a limited verification budget, the difficulty of the circuits is inversely proportional to the number of circuits that we can verify, $m = |\mathcal{V}|$. If the difficulty is too low, then the adversary can simulate the circuits with high fidelity close to the experimental fidelity, making it impossible to distinguish between classical and quantum samples even for large m. On the other hand, if the difficulty is too high, then m is too small, and the XEB score has significant fluctuations from experiment to experiment, and we cannot confidently distinguish between classical and quantum samples either in a given experiment. Therefore, the choice of m is crucial to the success of the protocol.

When planning the experiment, we first specify a target entropy (or Q) and the soundness parameter ε_{sou}. If the number of verification circuits m, the cost of simulating one circuit $\mathcal{B}$, and the classical computational power of the adversary $\mathcal{A}$ are given, we can determine the XEB score χ needed in order for the client to guarantee the target Q and ε_{sou}, which allows us to compute the honest case failure probability p_{fail}. However, even in the setting of fixed total verification budget $\mathcal{T}$, we have the freedom of choosing m as long as we adjust $\mathcal{B}$ appropriately as well, subject to $m = \lfloor \mathcal{T}/\mathcal{B} \rfloor$.

The trade-off between adversary simulation hardness and statistical uncertainties in the XEB score leads to an existence of an optimal m such that p_{fail} is minimal. Therefore, we optimize m to minimize p_{fail}. Mathematically, this amounts to the following:

$$\begin{aligned} \text{minimize} \quad & p_{\text{fail}}(m) = \Pr\left(\text{XEB}_{m,\phi} < \chi\right), \\ \text{such that} \quad & \varepsilon_{\text{adv}}(Q, \chi, m) = \varepsilon_{\text{sou}}. \end{aligned}$$

The failure probability $p_{\text{fail}}(m)$ is a function of m in a sense that $\Phi_{\mathcal{A}}$ in Eq. III.14 depends on $\mathcal{B} = \mathcal{T}/m$ and the honest server and adversarial CDFs in Eq. III.12 and III.20 explicitly depend on m as well. The $\Phi_{\mathcal{A}}$ dependence favors small m to decrease the adversarial classical fidelity. The explicit CDF dependence favors large m to reduce the statistical fluctuations. With the fixed total verification budget we possessed, with consideration of our circuit hardness and other protocol parameters, we verified $m = 1,522$ circuits.

C. Selection of Challenge Circuits

When choosing a circuit family for the challenge circuits, we must balance two considerations. On one hand, the circuit should be deep enough to make simulation difficult. On the other hand, the circuit should be shallow enough to enable high-fidelity execution on the quantum computer. Ref. [13] identifies a circuit family that achieves a high simulation cost while achieving very high fidelity on the H2-1 quantum processor. In this work we use the same circuit family. We refer the interested reader to Ref. [13] for a detailed discussion of the hardness of simulation of these circuits.

We specify the challenge circuits as follows. An n-qubit random circuit of depth d, $C_{n,d}$, consists of d layers of entangling gates. Each entangling layer is composed of a random set of $n/2$ disjoint $U_{\text{ZZ}}(\pi/2)$ gates, and each entangling layer is sandwiched by layers of random $SU(2)$ gates on all n qubits. U_{ZZ} is the native two-qubit gate on Quantinuum hardware. The arrangement of entangling operations in $C_{n,d}$ is obtained by solving the edge-coloring problem over a d-regular graph with n

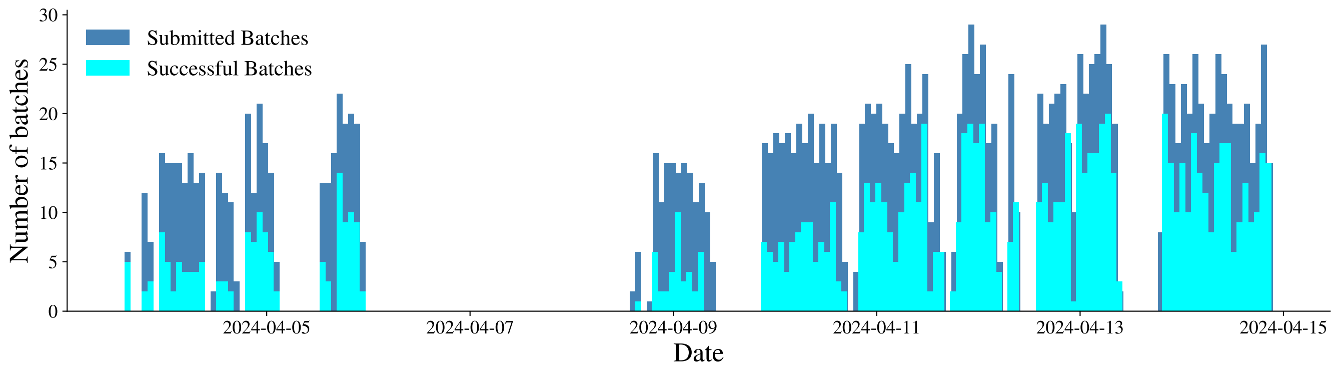

FIG. S7: Timeline of data collection

nodes, with each node representing a qubit. Assigning each layer to a color, the set of edges colored by a color $i \in [d]$ gives the disjoint qubit-pairings for entangling layer i. We refer to the arrangement of entangling pairs across all layers as a *topology*.

With a fixed circuit family, we must choose the depth d and a particular d-regular graph specifying the topology. For a fixed topology, the cost of performing exact tensor network contraction is independent of the choice of single-qubit gates. We generate numerous topologies with different d and calculate their "combo-128" costs. We then ignore topologies that are obviously too expensive or too cheap to simulate, and we estimate contraction time for the rest on the types of GPUs we expect to verify the circuits on. The details of the methods used to estimate the cost of simulation are given in Sec. IV A 3. This approach allows us to identify a $d = 10$ topology such that we can simulate close to m circuits with our total verification budget. With $n = 56$ and $d = 10$, these circuits have a total of $10 \times (56/2) = 280$ entangling gates and $11 \times 56 = 616$ single-qubit gates. As discussed earlier, once the circuit is chosen based on estimated costs from preliminary optimizations, ≈ 750k contraction schemes are then generated with different α to identify the scheme with the lowest time-to-solution on each supercomputer. This process lets us identify a *single* topology with the desired computational hardness and contraction schemes corresponding to the GPU models we use.

We generate challenge circuits by randomizing only the single-qubit $SU(2)$ gates over this chosen topology. In practice, this is achieved with finite randomness by discretizing the continuous group $SU(2)$. Crucially, the seed used to randomize single-qubit gates is kept secret.

D. Client-Server Interaction

Having fixed a topology, the client maintains a reservoir of circuits, where the single-qubit gates have been randomized using seeds kept secret from the server. These circuits are generated by using the commonly used library "Pytket" and are subsequently compiled to a H2-specific file type that spells out ion transport and gating operations necessary to implement the circuit in the H2-1 machine.

In total, we submit 60,952 circuits in 1,993 batches and receive 30,010 valid samples. The timeline of data collection is shown in Fig. S7. The t_{QC} for each circuit is taken to be the time interval recorded for that batch, divided by the size of the batch.

E. Verification

As discussed above, the final contraction schemes used by different supercomputers are obtained by choosing the lowest time-to-solution scheme from ≈ 750k schemes obtained by using KaHyPar, subtree reconfiguration, and simulated annealing provided by CoTenGra. For Frontier, CoTenGra with the JAX backend is used to perform contraction. For other supercomputers, cuQuantum is used to perform contraction, taking the contraction scheme obtained from CoTenGra as input. The details of techniques used to perform the exact contraction are given in Sec. IV A 3.

We remark that contraction of each slice is independent from each other, and the task is embarrassingly parallel. In Table III we show trivial scaling of the verification algorithm on all supercomputers. We also report the cost of contracting a single circuit in terms of the FLOP count, which is different between GPUs since the lowest time-to-solution contraction scheme is different between GPUs. Additionally, the full machines of Frontier and Summit were utilized, achieving numerical efficiencies of 45% and 59%, respectively. This translates to a sustained performance of 897 PFLOPS on Frontier and 228 PFLOPS Summit, which gives a total of 1.1 EFLOPS of sustained performance across both machines. The resulting time to simulate one circuit is 100.3 seconds on Frontier and 153.5 seconds on Summit.

	Single-GPU	10 nodes	100 nodes	1000 nodes	full-machine
Frontier	49%	45%	44%	45%	45%
Summit	64%	62%	61%	61%	59%
Perlmutter	60%	59%	59%	60%	N/A
Polaris	60%	60%	60%	N/A	N/A

TABLE III: Efficiency of tensor network contraction on different scales. We did not perform full-machine benchmarks on Perlmutter and Polaris. Additionally, Polaris has fewer than 1,000 nodes.

F. Randomness Extraction

The numbers corresponding to our experimental results are $n = 56, M = 30010, T_{\rm tot} = 64641$ seconds, $\mathrm{XEB}_{\rm test} = 0.32$, and $m = 1522$. The experiment passes the randomness extraction protocol with abortion thresholds $\chi = 0.30$ and $t_{\rm threshold} = 2.2$ s. For this protocol we first estimate the minimum number of $Q_{\min}$ against an adversary $\mathcal{A}$ for various values of $\varepsilon_{\rm sou}$ using Lemma 2 by performing binary search on Q such that $\varepsilon_{\rm adv}(Q, \chi) = \varepsilon_{\rm sou}$. Since Lemma 2 holds for any $\delta \geq 0$, we could optimize δ to minimize $\varepsilon_{\rm adv}$. In practice, we simply solve for δ such that $\varepsilon_1 = \varepsilon_{\rm adv}/2$.

We then determine the smooth min-entropy certified by our soundness result (Theorem 3 for $Q_{\min}$ samples, with smoothing parameter $\varepsilon_{\rm s} = \varepsilon_{\rm sou}/4$). Table I in the main text reports the smooth min-entropy rate $h = H_{\min}^{\varepsilon_{\rm s}}/(M \cdot n)$. For example, at $\varepsilon_{\rm sou} = 10^{-6}$ and $\mathcal{A} = 4\times$Frontier, we have $h = 0.04$.

Having determined the certified entropy present in the M samples, we can pass the raw bits into a randomness extractor. The length of the extractor output is given by Corollary 7. We feed a total of $56 \cdot M$ bits into a seeded randomness extractor to obtain ℓ bits, which are now expected to be close to the uniform distribution. In this work we use the Toeplitz extractor, which is known to be a quantum-proof strong extractor [45]. A property of a strong extractor is that the extractor output is independent of the seed used. If the client uses randomness privately, the net output is the concatenation of the extraction seed and the output of the extractor. We use the implementation of the Toeplitz extractor from the open-source package "Cryptomite" [33].

V. Details on Outlook for Future Experiments

We would like to understand how improvements in the quantum computer fidelity, average time per sample, and verification budget improve the protocol performance. We are interested in the change in the resulting smooth-min-entropy as well as achievable security parameters. For the protocol to be economically viable, only a very small fraction of samples can be verified ($m/M \ll 1$). In the limit of infinite M and finite m, we can lower bound the fraction Q/M of quantum samples $R_{\rm Q}$ instead of the absolute number of quantum samples, and the normalized entropy is $h = R_{\rm Q} \times (n-1)/n$. The average classical simulation fidelity is

$$\langle \phi_{\mathcal{A}} \rangle = \min\left(1, \frac{c_{\rm eff} \cdot \mathcal{A} \cdot \langle t_{\rm threshold} \rangle}{(1-R_{\rm Q}) \cdot \mathcal{B}}\right) = \min\left(1, \frac{c_{\rm eff} \cdot \mathcal{A} \cdot \langle t_{\rm threshold} \rangle}{(1-R_{\rm Q}) \cdot \mathcal{T}/m}\right). \tag{V.1}$$

In the limit of infinite M and finite m, there is no uncertainty in the fraction R of Porter–Thomas bitstrings (as can be seen in Eq. III.17 since $\mathbb{E}[L_{\rm C}] \to \infty$), and we have $R = R_{\rm Q} + (1 - R_{\rm Q}) \cdot \langle \phi_{\mathcal{A}} \rangle$. Therefore, the number of Porter–Thomas bitstrings in the verification set follows the binomial distribution, and we have

$$\Pr\left(\mathrm{XEB}_{\rm test} \leq \chi\right) = \sum_{l=0}^{m} \Pr\left(\mathrm{XEB}_{\rm test} \leq \chi | l \text{ PT bitstrings in } \mathcal{V}\right) \cdot \Pr\left(l \text{ PT bitstrings in } \mathcal{V}\right) \tag{V.2}$$

$$= \sum_{l=0}^{m} \tilde{\Gamma}(m+l, m \cdot (\chi+1)) \cdot \left[\binom{m}{l} R^l (1-R)^{m-l}\right], \tag{V.3}$$

which is the same as the CDF for a finite-fidelity honest server with fidelity R.

Now, we would like to understand the effect of improving the quantum computer fidelity ϕ, decreasing the threshold of average time per sample $t_{\rm threshold}$, increasing the verification budget $\mathcal{B}$, and decreasing the adversary computational power $\mathcal{A}$. Further, as discussed in Sec. III D, $t_{\rm threshold}$, $\mathcal{B}$, and $\mathcal{A}$ can be treated interchangeably since they only affect the CDF calculations by entering $\Phi_{\mathcal{A}}$ in Eq. III.14. For each set of parameters, we can similarly optimize m following the procedure in Sec. IV B. This allows us to map out the landscape of achievable performance in Fig. 3 of the main text.

VI. Table of Variables

Label	Meaning
n	Number of qubits
$\mathcal{B}$	Cost of simulating challenge circuits
$\mathcal{T}$	Total classical computational budget for verification
M	Number of successful samples
r	Length of the circuit generation seed
K_{ext}	Seed for the randomness extractor
x_i	Bitstring for the ith circuit
X^M	Client's classical register of the received M samples
K	Client's output register
b	Number of stitched circuits per job, where each stitched circuits is composed of 2 circuits
$T_{b,\text{cutoff}}$	Cutoff time for a single batch of $2b$ circuits
T_{tot}	Total time for all successful batches
t_{QC}	Average response time per successful quantum sample
$t_{\text{threshold}}$	Target for average time per successful quantum sample for protocol abort
$T_{\text{threshold}}$	$M \cdot t_{\text{threshold}}$
$\mathcal{V}$	The set of indices for circuits used for verification
m	The size of the verification set
XEB_{test}	The XEB score of the verification set
χ	XEB score threshold
m	Number of samples used in XEB
ϕ	Expected fidelity of H2-1 on challenge circuits
p_{fail}	Abort probability for an honest server
ε_{sou}	Soundness parameter
$\varepsilon_{\text{accept}}$	Probability of the protocol not aborting when interacting with an adversary
ε_{s}	Min-entropy smoothing parameter
U	Client
S	Server
Ξ	Server controller
S_{Q}	Server quantum computer
S_{C}	Server classical computer
I_{sn}	Initial snapshot of all classical information of the client and the server
$\mathcal{A}$	Adversary classical computational power in FLOPS
c_{eff}	Numerical efficiency of tensor network contraction
Q	Number of quantum samples from the adversary
Q_{min}	Minimum number of quantum samples the adversary must generate to pass the XEB test with probability at least $\varepsilon_{\text{accept}}$
M'	Number of amplitudes to compute for frugal rejection sampling by the adversary
$\phi_{\mathcal{A}}^{(i)}$	Adversary's classical simulation fidelity of the ith accepted circuit
$\Phi_{\mathcal{A}}$	Sum of classical simulation fidelity for an adversary with computational power $\mathcal{A}$
$\langle \phi_{\mathcal{A}} \rangle$	Average classical simulation fidelity for an adversary with computational power $\mathcal{A}$ in the limit of infinitely many samples
$\mathcal{U}$	Uniform distribution
$\mathcal{H}$	Ensemble of Haar-random states

TABLE IV: Summary of experimental parameters used in this work.

[1] S. Aaronson and S.-H. Hung, Proceedings of the 55th Annual ACM Symposium on Theory of Computing , 933 (2023).
[2] S. Aaronson and S.-H. Hung, Preprint at https://arxiv.org/abs/2303.01625 (2023).
[3] F. Arute et al., Nature **574**, 505 (2019).
[4] S. Goldwasser and M. Sipser, in Proceedings of the Eighteenth Annual ACM Symposium on Theory of Computing, STOC '86 (Association for Computing Machinery, New York, NY, USA, 1986) pp. 59–68.

[5] F. Dupuis, O. Fawzi, and R. Renner, Communications in Mathematical Physics **379**, 867 (2020).
[6] R. Bassirian, A. Bouland, B. Fefferman, S. Gunn, and A. Tal, Preprint at https://arxiv.org/abs/2111.14846 (2021).
[7] Y. Liu et al., Phys. Rev. Lett. **132**, 030601 (2024).
[8] S. Boixo et al., Nat. Phys. **14**, 595 (2018).
[9] "NIST digital library of mathematical functions." https://dlmf.nist.gov/8.2, accessed: 2024-07-26.
[10] N. T. Thomopoulos, Statistical Distributions (Springer International Publishing, 2017).
[11] A. Morvan et al., Nature **634**, 328–333 (2024).
[12] Q. Zhu et al., Sci. Bull. **67**, 240 (2022).
[13] M. DeCross et al., Preprint at https://arxiv.org/abs/2406.02501 (2024).
[14] S. Aaronson and L. Chen, Proceedings of the 32nd Computational Complexity Conference , 1 (2017).
[15] S. Aaronson and S. Gunn, Theory Comput. **16**, 1 (2020).
[16] A. Bouland, B. Fefferman, C. Nirkhe, and U. Vazirani, Nat. Phys. **15**, 159 (2018).
[17] A. Bouland, B. Fefferman, Z. Landau, and Y. Liu, in 2021 IEEE 62nd Annual Symposium on Foundations of Computer Science (FOCS) (IEEE, 2022).
[18] H. Krovi, arXiv preprint arXiv:2206.05642 (2022).
[19] X. Gao et al., PRX Quantum **5**, 010334 (2024).
[20] B. Ware et al., Preprint at https://arxiv.org/abs/2305.04954 (2023).
[21] D. Aharonov, X. Gao, Z. Landau, Y. Liu, and U. Vazirani, in Proceedings of the 55th Annual ACM Symposium on Theory of Computing (2023) pp. 945–957.
[22] I. L. Markov, A. Fatima, S. V. Isakov, and S. Boixo, "Quantum supremacy is both closer and farther than it appears," (2018).
[23] X.-H. Zhao et al., Preprint at https://arxiv.org/abs/2406.18889 (2024).
[24] L. T. A. N. Brandão and R. Peralta, NIST White Paper (2020).
[25] R. Renner, Int. J. Quantum Inf. **6**, 1 (2008).
[26] M. Tomamichel and A. Leverrier, Quantum **1**, 14 (2017).
[27] M. Tomamichel, Quantum Information Processing with Finite Resources: Mathematical Foundations, 1st ed. (Springer Publishing Company, Incorporated, 2015).
[28] T. Hagerup and C. Rüb, Inf. Process. Lett. **33**, 305–308 (1990).
[29] J. A. Rice, Mathematical Statistics and Data Analysis, Vol. 371 (Thomson/Brooks/Cole Belmont, CA, 2007).
[30] M. Tomamichel, R. Colbeck, and R. Renner, IEEE Transactions on Information Theory **55**, 5840 (2009).
[31] R. Konig and R. Renner, IEEE Transactions on Information Theory **57**, 4760 (2011).
[32] A. De, C. Portmann, T. Vidick, and R. Renner, SIAM J. Comput. **41**, 915 (2012).
[33] C. Foreman, R. Yeung, A. Edgington, and F. J. Curchod, Preprint at https://arxiv.org/abs/2402.09481 (2024).
[34] E. Stoudenmire and D. J. Schwab, in Advances in Neural Information Processing Systems, Vol. 29, edited by D. Lee, M. Sugiyama, U. Luxburg, I. Guyon, and R. Garnett (Curran Associates, Inc., 2016).
[35] R. Orús, Nat. Rev. Phys. **1**, 538 (2019).
[36] J. Chen, F. Zhang, C. Huang, M. Newman, and Y. Shi, "Classical simulation of intermediate-size quantum circuits," (2018).
[37] Y. Liu et al., in Proceedings of the International Conference for High Performance Computing, Networking, Storage and Analysis, SC '21 (ACM, 2021).
[38] Y. a. Chen, in Proceedings of the 28th ACM SIGPLAN Annual Symposium on Principles and Practice of Parallel Programming, PPoPP '23 (ACM, 2023).
[39] J. Gray and S. Kourtis, Quantum **5**, 410 (2021).
[40] "Frontier user guide," https://docs.olcf.ornl.gov/systems/frontier_user_guide.html, accessed: 2024-07-26.
[41] "Summit user guide," https://docs.olcf.ornl.gov/systems/summit_user_guide.html, accessed: 2024-07-26.
[42] "Perlmutter architecture," https://docs.nersc.gov/systems/perlmutter/architecture/, accessed: 2024-04-24.
[43] "Polaris machine overview," https://docs.alcf.anl.gov/polaris/hardware-overview/machine-overview/, accessed: 2024-07-26.
[44] "TOP500 website," https://www.top500.org/lists/, accessed: 2024-07-26.
[45] R. Konig, U. Maurer, and R. Renner, IEEE Transactions on Information Theory **51**, 2391 (2005).
